# A Comprehensive Review on Collagen Type I Development of Biomaterials for Tissue Engineering: From Biosynthesis to Bioscaffold

**DOI:** 10.3390/biomedicines10092307

**Published:** 2022-09-16

**Authors:** Ibrahim N. Amirrah, Yogeswaran Lokanathan, Izzat Zulkiflee, M. F. Mohd Razip Wee, Antonella Motta, Mh Busra Fauzi

**Affiliations:** 1 Centre for Tissue Engineering and Regenerative Medicine, Faculty of Medicine, Universiti Kebangsaan Malaysia, Jalan Yaacob Latiff, Bandar Tun Razak, Cheras, Kuala Lumpur 56000, Malaysia; 2 Institute of Microengineering and Nanoelectronics (IMEN), Universiti Kebangsaan Malaysia, Bangi 43600, Malaysia; 3 Department of Industrial Engineering, University of Trento, Via Sommarive 9, 38122 Trento, Italy

**Keywords:** collagen, tissue engineering, scaffold, biomaterial, purification, fabrication

## Abstract

Collagen is the most abundant structural protein found in humans and mammals, particularly in the extracellular matrix (ECM). Its primary function is to hold the body together. The collagen superfamily of proteins includes over 20 types that have been identified. Yet, collagen type I is the major component in many tissues and can be extracted as a natural biomaterial for various medical and biological purposes. Collagen has multiple advantageous characteristics, including varied sources, biocompatibility, sustainability, low immunogenicity, porosity, and biodegradability. As such, collagen-type-I-based bioscaffolds have been widely used in tissue engineering. Biomaterials based on collagen type I can also be modified to improve their functions, such as by crosslinking to strengthen the mechanical property or adding biochemical factors to enhance their biological activity. This review discusses the complexities of collagen type I structure, biosynthesis, sources for collagen derivatives, methods of isolation and purification, physicochemical characteristics, and the current development of collagen-type-I-based scaffolds in tissue engineering applications. The advancement of additional novel tissue engineered bioproducts with refined techniques and continuous biomaterial augmentation is facilitated by understanding the conventional design and application of biomaterials based on collagen type I.

## 1. Introduction

As part of regenerative medicine, the tissue engineering field aims to repair or replace damaged, diseased, or lost tissues or organs by using bioengineering to design suitable bioconstructs in vitro or transplanted in vivo [1]. The idea is to fabricate biomaterials that can maintain, enhance, or restore functionality. The extracellular matrix (ECM) serves as the connective tissue that provides not only physical support for cell arrangement but also the space for all the extracellular molecules, such as growth factors and cytokines, for appropriate signalling mechanisms to have normal tissue function [2]. Therefore, scaffolds that can mimic the ECM are fundamental in tissue engineering, along with cellular compatibility and the ability for full tissue regeneration.

As the main protein content, collagen is significant for the maintenance of biological and structural integrity in the ECM, and it is highly dynamic due to the constant remodelling based on proper physiological functions. Collagens account for 30% of the total body protein content in humans. They are a group of proteins with a specific molecular structure and at least some of them possess fibrillary structures, which are essential for tissue scaffolding to perform its normal functions and provide major tensile strength to the ECM. The various roles of collagens include aiding cell adhesion and migration, tissue scaffolding, and tissue repair [3]. There are 28 types of collagen that have been identified and well characterised [4]. However, the class of collagen representing more than 90% of human collagen is fibrillar collagen, which can form fibrils, which includes types I, II, III, V, and XI. The fibrillary component contributes to providing tensile strength, maintaining the stability of tissues, and preserving structural integrity. Other common collagens, such as type IV and VII, construct the network structure of basement membranes [5]. Collagen type I is expressed in virtually all ECM and connective tissues in the human body. Type II is mostly in the cartilage, type III is in the skin, blood vessels, and organs to a lesser extent, and type V is in the tissue cytoskeleton.

Thus, collagen type I is the most abundant type of collagen with its most prominent functional roles in the skin and bone, and to a lesser extent in the ECM of other tissues [6]. In the skin, collagen type I constitutes 80–85% of the dermal ECM, while collagen type III constitutes about 8–11% [7]. Bone tissues have more than 90% collagen type I with a small quantity of type V collagen with hydroxyapatite (HA) crystals anchored by the collagen framework [8]. Tendon is made up of 70% water and 30% of dry mass, of which 60–80% is collagen type I and 2% is elastin [9]. To a much lesser degree, collagen types III and V are also represented in the tendon. For cartilage, collagen makes up about 60% of its dry weight. However, collagen type II represents 90–95% of the ECM cartilage, and while collagen types I, IV, V, VI, IX, and XI are present, they play a minor role in the formation and stabilisation of the type II fibril network [10]. Nevertheless, cartilage tissue engineering widely uses type I collagen scaffolds with some bioproducts undergoing clinical trials due to its ubiquitous biocompatibility and large-scale clinical approval [11]. For the cornea, collagen type I consists of 80–90% of the fibrillar collagen from corneal keratocytes, and while collagen type V is present, it is quantitatively minor [12].

To date, much profound research on collagen type I’s properties has strengthened knowledge and is beneficial for diverse therapeutic applications and tissue engineering [13]. It is a natural biomaterial and is commonly used in the formation of tissue substitutes, controllable drug release (smart scaffolds), wound dressings, the substrate of fragrances and cosmetic enhancement therapy, and the treatment of related medical diseases such as bone regeneration [14,15,16,17,18,19]. Due to its advanced binding capacity, collagen could also benefit the application of drug delivery systems, growth factors, or cell carriers [20]. Their versatility and mechanical properties compel the use of type I collagen for multi-purpose bioscaffold designs.

**Figure 1:**
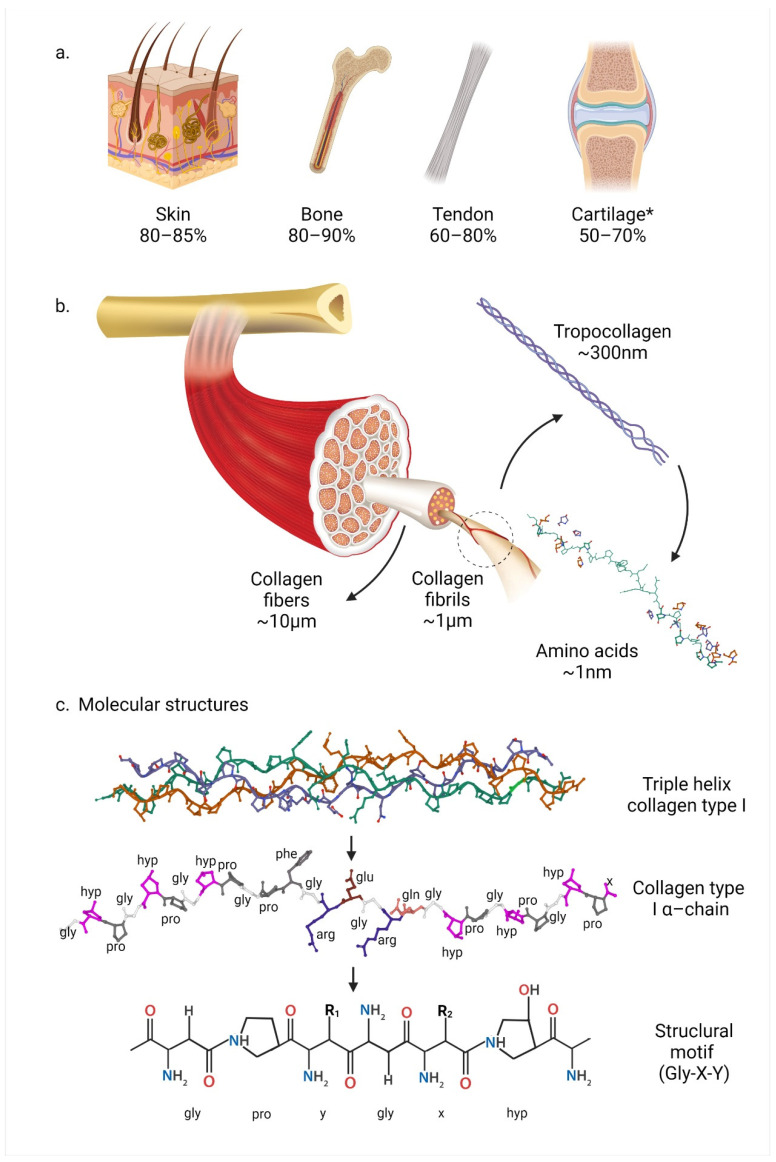
(**a**) Collagen type I sources and approximate content in each tissue. * Cartilage percentage is based on all collagens in their dry weight, as it is predominantly collagen type II. (**b**) Collagen fibre structure, fibrils, triple helices of alpha chains as tropocollagen, and amino acid residues. (**c**) Triple-helix and single-chain collagen type I molecular structure (PDB 1CAG) [24] with gly-x-y repeats; x-y typically represents proline and hydroxyproline, respectively.

Biomaterials are intended to be products that can replace native collagen-based ECM. The characteristics and composition of biomaterials applied as scaffolds for tissue engineering have an important impact on the conditions for collagen engineering as well as the regeneration of neo-tissues. However, collagen comes with a myriad of complexities in its characteristics, types, fibril arrangement, and structure-related functions. Tissues that are generally involved in collagen-based scaffolds include skin, bone, tendon, cartilage, and the cornea, which many scientific investigations have reported. Currently, type I collagen is being used in research to produce biomaterials originating from various sources such as mammalian, amphibian, fish, marine, bird, and human recombinant collagen [21], which will be discussed below. Figure 1a illustrates the approximate type I collagen content in specific tissues that are isolated from the skin, bone, tendon, and cartilage. The clinical potential of these collagens is gaining precedence, particularly with bone and skin tissue [22,23]. Many novel collagen-type-I-based biocomposites are in the proof-of-concept stage. This review will discuss the functions and applications of collagen type I in the tissue engineering field. The collagen type I structure, biosynthesis, sources, isolation techniques, and characterisation of the physicochemical properties of the fabricated materials are also included for the fundamental understanding of collagen engineering.

## 2. Molecular Structure and Biosynthesis of Collagen Type I

### 2.1. Types of Collagens

The collagen superfamily, with 28 identified thus far, is further divided into eight subfamilies whereby the majority of collagen types belong to the fibrillar subfamily or fibril-forming, such as type I collagen. Based on their fibre architecture, collagens are classified into fibril-forming collagens, anchoring collagens, network-forming collagens, transmembrane collagens, and fibril-associated collagens with interrupted triple helices (FACIT) [25]. Collagen type is determined by the complexity and diversity of collagen structure, non-helical domains, assembly function, and splice variations. Types I, II, III, V, and IX, also known as fibril-forming collagens, align into giant fibrils. Type IV creates an interlacing network in the basement membranes, while type VI forms microfibrils, and type VII forms anchoring fibrils. Types IX, XII, XIV, XIX, XX, and XXI of FACIT collagens are connected with bigger collagen fibres, which operate as molecular bridges to support ECM organisation. Collagen type I makes up about 80% of total collagen and is found in abundance in the human dermis.

### 2.2. Collagen Type I Supramolecular Structure

Figure 1b illustrates the collagen structure from tissue to polypeptides. The molecular structure of collagen is defined by three polypeptide α-chains, which form a triple helix. Single α-chains occur in a left-handed polyproline-II-type helix. These polypeptides then coil around each other, forming a right-handed triple helix, which is also known as tropocollagen. The collagen triple helix can be formed by identical α-chains (homotrimers) or different α-chains (heterotrimers). Collagen types I, IV, V, VI, IX, and XI are heterotrimers, whereas collagen types II, III, VII, VIII, and X are all homotrimers [7]. In collagen type I, the α-chain polypeptides consist of two exact chains of alpha-1 (α1), and the third is alpha-2 (α2), which are joined into a triple helix, making it a heterotrimer (α1(I)2–α2(I)). The collagen triple helices interact and assemble into microfibrils, stabilised mainly by hydrogen bonding, resulting in supramolecular structures [26]. Various polypeptide chain combinations would result in different collagen properties. The 28 different collagen types were led by the discovery of 25 α-chains [7].

At the next hierarchical level, collagen microfibrils then assemble into supramolecular complexes of fibrillar collagen, forming the elongated fibrils of collagen with different diameters dependent on where the tissues or organs contain fibrils [27]. Generally, the supramolecular structure of the collagen type I conformation has overlapping bands of 67 nm with each molecule being 35–500 nm long, depending on the localisation and a 40 nm gap between successive macromolecules [28]. This remarkable property has been used to characterise the banding pattern of collagen fibrils by using scanning electron microscopy (SEM), transmission electron microscopy (TEM), atomic force microscopy (AFM), and X-ray diffraction (XRD) investigations [29,30,31,32]. Depending on the arrangement of the collagen monomers, a banding pattern with a periodicity of 70 nm is typically observed [33]. Any changes to the arrangement could result in a different periodicity value. The collagen fibrils then build into organised structures with particular morphologies for specific tissues. The organisation of the collagen fibres to form and also to support connective tissues is the resulting tissue function.

Tissue engineering relies on structural data in order to mimic the ECM as closely as possible for the targeted tissue. For human skin, a team in 2019 used multiphoton microscopy, a technique to observe living tissue in fine detail. The team found evidence that type I collagen forms a complex network of interlacing fibrils in a mesh-like lattice, which contradicts the clear geometric orientation that was previously hypothesised [34]. In addition, they observed a novel breakthrough in which the elastin fibres of the connective tissue also follow the same orientation. The dynamic changes in collagen structure within the same tissue were also found, whereby the collagen fibres in the deep reticular dermis had a larger mean horizontal diameter compared to the superficial reticular dermis (47 ± 18 µm vs. 136 ± 14 µm; *p* < 0.01) [34].

In bone, the collagen network with HA crystals forms a highly complex and closely interwoven, but ordered composite, which is organised into lamellae, or layers, which are only a few microns thick. The collagen framework is predominantly collagen type I fibrils in concentric weaves of elaborate 3D arrays, and the anchored HA crystals are 28 nm wide, 50 nm long, and 2 nm thick [26]. In bioengineered bones, polymerisation, fibrillogenesis, and fibre development are well-known processes that are further modified to create various forms of collagen scaffolds for numerous bone tissue engineering applications [19]. For example, the degree of collagen crosslinking and the composite of organic mineral remodelling processes may adjust the mechanical properties by varying fibril orientation as the collagen fibres are the principal source of the tensile strength of bone tissues [2].

In matured tendons, larger-diameter fibrils of 500 nm align in parallel bundles to support their biological demands for high tensile strength [9]. In the cornea, smaller-diameter (20 nm) collagen fibrils are arranged orthogonally to retain optical transparency while maintaining their structure [35]. In addition, collagen fibres in cartilage are packed differently based on their zonal architecture, which requires different functions. Collagen fibres, primarily types II and IX, are packed tightly with parallel alignment to the articular surface in the thin superficial (tangential) zone and have a high number of flattened chondrocytes. The arrangement ensures that the tangential zone can perform its main function of protecting and maintaining the deeper layers. The middle (transitional) zone has thicker collagen fibres, which are organised obliquely with the addition of proteoglycans, while the available chondrocytes are spherical and low density. The transitional zone aims to provide the initial resistance to compressive forces, hence the addition of proteoglycans. The deep zone of cartilage is meant to provide the highest resistance to compressive forces. As such, they have the largest diameter of collagen fibrils arranged perpendicular to the articular surface and are in a radial disposition. The chondrocytes are organised into a columnar orientation parallel to the collagen fibres and perpendicular to the joint line. To have high resistance, the deep zone must have the highest proteoglycan content with the lowest water concentration [10]. Thus, the collagen structure is aligned according to the needs of the local tissue.

To date, collagen type I has received the most attention in the study of collagen structure and function. The biological functions of collagen are drawn from its repetitive primary structure, its ability for coordinated self-assembly, and its local microenvironment requirements. With this information, collagen-type-I-based biomaterials for regeneration should provide collagen scaffolds resembling the native tissue collagen organisation and content. Furthermore, understanding the reversible nature of the hierarchical self-assembly of collagen led to the development of tissue engineering whereby biopolymers and supramolecular polymers of collagen derivatives are used for biomedical applications rather than macromolecules [36]. Designing biopolymers is advantageous in biomedical applications due to their ability to imitate the ECM in both architecture and cell signalling capacity.

### 2.3. Collagen Type I Molecular Structure and Content

Figure 1c depicts the collagen molecular structures. At a molecular level, collagens have a repeated proline-rich amino acid sequence, with a glycine (Gly) residue appearing at every third position to form the triple helix. Moreover, 4-hydroxyproline is generally present from a posttranslational modification of peptide-bound prolyl residues, which also provide a distinctive marker of these molecules [37]. The repeated sequence is the Gly-X-Y motif in which X and Y can be any amino acid, although they are most commonly found as proline and 4-hydroxyproline, which are necessary for collagen’s gelling function [38]. The length of the triple helix structure varies between collagen types, resulting in a 300 nm structure that corresponds to 1000 amino acids with a repeated Gly-X-Y pattern. Gly residues form a central axis in the triple helix, positioning the other X and Y residues on the helix’s surface, thus making them accessible for binding interactions, which are vital in the intermolecular interactions of fibril formation [39].

Although the amino acid compositions of collagen types vary between species, the common elements in the collagen fibril structure at the molecular level, with similarities in the distinctive amino acid sequences, are impressive characteristics. These similarities provide the groundwork for various tissue engineering developments that share common chemical and physical properties. All collagen molecules contain hydroxyproline, and along with glycine and proline, these three account for more than 50% of the total amino acid content [26]. Amino acid sequences play a vital role in determining the 3D assembly conformations, and adding other amino acids could be used to distinguish different collagen types. Most mammalian collagen contains much hydroxyproline and hydroxylysine, as well as much proline and hydroxyproline. Poultry collagen has a similar or slightly lower imino acid content than mammalian collagen, whereas fish collagen has a lower imino content, but higher serine, methionine, and threonine [40]. An imino acid molecule has both imine (>C=NH) and carboxyl (-C(=O)-OH) functional groups. These variations in imino acid can affect chemical and physical properties, thermal stability, viscosity, and the degree of crosslinking [7,41], which are all important to address when developing suitable biomaterials for tissue engineering applications.

### 2.4. Biosynthesis

**Figure 2:**
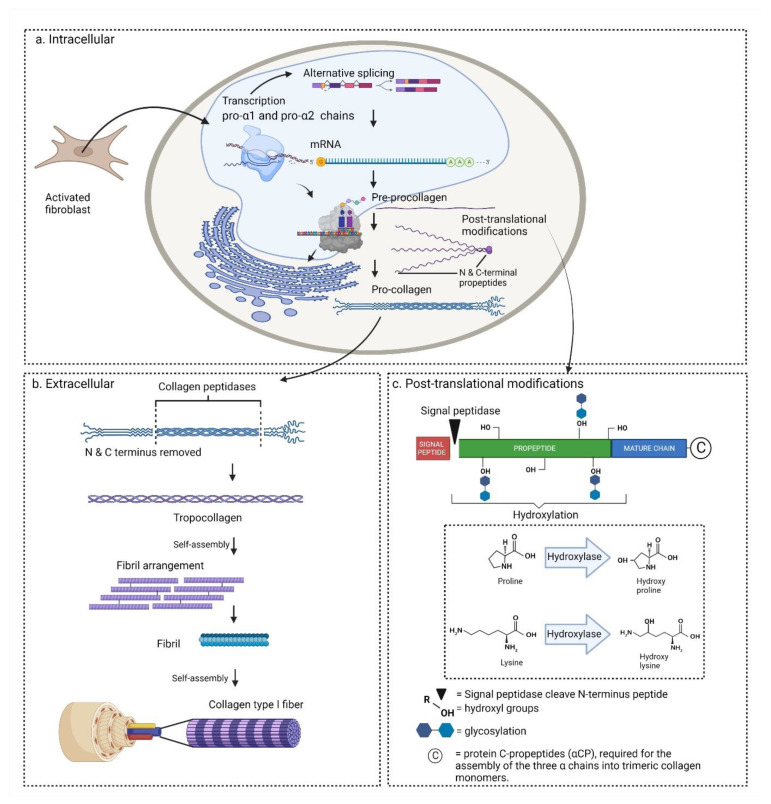
Overview of collagen type I biosynthesis from (**a**) intracellular transcription to (**b**) formation of extracellular complex macromolecular structures and what happens in between during (**c**) post-translational modifications.

Collagen production is a complex process that includes everything from gene transcription in the nucleus of cells to the formation of heterotrimeric structures and fibrils. Collagen type I is synthesised both intracellularly and extracellularly. Figure 2 depicts an overview of collagen biosynthesis. Briefly, the biological synthesis, secretion, and assembly of collagen can be described as follows [42]. After transcription in the nucleus, the single polypeptide chains, called pre-procollagen chains, are translated into the membrane-bound ribosomes. The chain has three major domains, namely the α-chain, the amino-terminal peptide, and the carboxy-terminal peptide. In the rough endoplasmic reticulum (RER), three pro-α chains affiliate and become a procollagen molecule with a triple helix structure. Amid this process, the pre-peptide sequences are removed, the hydroxylation of lysine and proline residues occurs, the pre-peptides become glycosylated, and disulphide bonding occurs. After these post-translational modifications, individual procollagen molecules are transported into the Golgi complex, packaged into granules, and secreted into the extracellular space. For collagen type I, the extension peptides at both ends are cleaved before the fibres can be assembled. After this, several collagen molecules interact in a quarter-staggered manner and assemble into collagen fibrils, and subsequently, collagen fibres and bundles are formed. The details of these processes are described below.

#### 2.4.1. Transcription and Translation

Fibroblasts are responsible for the majority of collagen synthesis. On the other hand, collagens have tissue specificity and are also synthesised by keratinocytes, smooth muscle cells, and vascular endothelial cells. Although the cell type largely determines the regulation of collagen transcriptional activities, it can also be influenced by a variety of growth factors and cytokines. Normally, collagen gene expression is low and steady, but during wound healing, collagen transcription rates are increased to initiate tissue repair. However, if it is unable to return to basal rates in a timely and appropriate manner, the over-production may result in abnormal scar formation and fibrosis. In cell fibrosis or wound healing, collagen type I production can be increased by several hundred-fold due to the post-transcriptional regulations, such as the increased half-life of the mRNA, resulting in increased translatability [43].

The genes for pro-α1 and pro-α2 are transcribed during transcription (Figure 2a). Most collagen genes have a complex exon–intron pattern ranging from 3 to 117 exons, and fibrillary collagen mRNAs are encoded by more than 50 exons [44]. For human collagen type I, the α1 chain is coded by 41 exons with two joining exons for telopeptides and Gly-X-Y repeats characteristic of the triple helical domain, while the α2 chain is coded by 42 exons with two joining exons [28]. Due to the multiple transcription initiation sites and alternative splicing exons at 3′ or 5′ or both, mRNA of various sizes can be detected in a large portion of collagen transcription. Furthermore, before the mature mRNA is transported to the cytoplasm and interacts with ribosomes for translation, the pre-mRNA undergoes capping at the 5’ end and polyadenylation at the 3’ end.

Ribosome-bound mRNA is translated into pre-procollagen molecules, which are then transported to the RER for post-translational modification. These early precursors distend into the RER lumen, aided by a signal recognition domain, and are recognised by the corresponding receptors [44]. The polypeptides are modified co-translationally, and the rate of modification is dynamically equilibrated with the rate of folding, thus suggesting coordinated translation of collagen α1(I) and α2(I) polypeptides [43].

#### 2.4.2. Post-Translational Modifications

Pre-procollagen polypeptides undergo post-translational processing in three major modifications in the RER to synthesise fibrillar collagen in its precursor form, which is procollagens (Figure 2c).

First, the N-terminal signal peptide is removed. Following the removal of the signal peptide by a signal peptidase, the procollagen molecules go through a series of post-translational modifications. The 3′ untranslated region (UTR) of collagen α1(I)l mRNA contains a C-rich sequence that binds to protein C-propeptides (αCP), which is important in stabilising the mRNA to produce collagen. The αCP is required for the assembly of the three alpha chains into the trimeric collagen monomers. The αCP’s globular structure is stabilised by intra-chain disulphide bonds, and the oligosaccharyl transferase complex adds an N-linked carbohydrate group. The formation of triple helices begins with the alignment of the C-terminal domains of three α-chains, and this process initiates the formation of the triple helix, thus progressing to the N-terminus [45,46].

Second, depending on the collagen type, post-translational enzymatic hydroxylation on specific proline and lysine occurs [47]. The formation of intramolecular hydrogen bonds in the presence of 4-hydroxyproline is required for the stability of the triple helix structure [48]. Hydroxylation of proline and lysine residues is catalysed by prolyl 3-hydroxylase, prolyl 4-hydroxylase, and lysyl hydroxylase [47]. All three enzymes require ferrous ions, 2-oxoglutarate, molecular oxygen, and ascorbate (vitamin C) as cofactors [28]. In fibril-forming collagens, approximately 50% of the proline residues have a hydroxyl group at position 4, and the extent of prolyl-hydroxylation varies by species; for example, colder climate organisms have less hydroxylation [49]. The presence of 4-hydroxyproline is required for intramolecular hydrogen bonds, which contributes to the thermal stability of the triple helical domain and, thus, to the monomer and collagen fibril integrity [44]. Hydroxylysine residues can facilitate stable intermolecular crosslinking of collagen molecules in fibrils and also serve as sites for carbohydrate attachment.

Third, after adding hydroxyl groups to lysine and proline residues, glycosylation of the selected hydroxyl groups with galactose and glucose b occurs on lysine. Glucosyl- and galactosyl residues are transferred to the hydroxyl groups of hydroxylysine by the hydroxylysyl galactosyltransferase and galactosyl-hydroxylysyl-glucosyltransferase enzymes [50]. Thus, by zipper-like folding, the hydroxylated and glycosylated pro-α chains assemble from a left-handed triple helix into a right-handed coil and form procollagens.

#### 2.4.3. Collagen Secretion

Following processing and procollagen assembly, the triple helical molecules are packed into secretory vesicles within the Golgi compartment and released into the extracellular space as a propeptide. Following secretion, the procollagen trimers are processed based on the type of collagen. However, in general, collagen peptidases conduct propeptide cleavage and remove the ends of the procollagen, resulting in tropocollagens. Human procollagen type I N-propeptides and C-propeptides are cleaved off by a group of metalloproteinases, namely the N-protease ADAMTS and the C-protease BMP1, respectively, to form tropocollagen, which is a mature collagen type I [28,47]. ADAMTS is a disintegrin and metalloproteinase with thrombospondin motifs, while BMP1 and tolloid-like proteinases are bone morphogenetic proteins [46,47].

Tropocollagen exists as a monomer and undergoes supramolecular organisation. The strength of the longitudinal assembly is derived from covalent parallel intramolecular and intermolecular crosslinking into fibrils and multimeric fibres (Figure 2b). Covalent crosslinks in collagen type I are typically formed from lysine and hydroxylysine residues, which are aided by lysyl oxidase, a copper-dependent enzyme. However, cysteine-derived disulphide bonds can be formed in some collagens. These covalent crosslinking bonds form strong collagen fibrils, which can then be assembled into fibres.

#### 2.4.4. Clinical Significance of Biosynthesis for Tissue Engineering

The complexities of collagen biosynthesis and assembly steps are tightly regulated and executed. Type I collagen binds to type III collagen to form broad extracellular fibres in the dermis. Mutations in the collagen or processing enzymes can cause connective tissue abnormalities, such as various forms of Ehlers-Danlos Syndrome (EDS), and mutations in the collagen type I gene can cause osteogenesis imperfecta [51]. In addition, nutritional deficiencies can also affect enzymatic function. For example, the hydroxylase enzyme function that adds hydroxyl groups to lysine and proline, which is necessary for intramolecular hydrogen bonds, uses vitamin C as a cofactor. Vitamin C deficiency causes scurvy (ascorbic acid levels of less than 11 micromoles/L), and although it is rare in the developing world, malnutrition is at risk among infants, the elderly, and alcoholics [42]. Fortunately, treatment is available in the form of supplementation and diet.

Osteogenesis imperfecta is a family of genetic disorders that make bones weak and easily breakable. About 85% of patients have mutations in the *COL1A1* or *COL1A2* genes for type I collagen. However, many other genes related to type I collagen have been described, such as enzymes that modify collagen type I, chaperone proteins, and signalling proteins [52]. The types are categorised based on the severity, which includes type I (mild), type II (perinatally lethal), type III (severe), and type IV (moderate) [53]. The conventional treatments include surgery and physiotherapy in addition to pharmacological approaches such as bisphosphonates (antiresorptive agents), neridronate (growth hormone increased bone density), teriparatide (anabolic agent stimulating bone formation), denosaub (monoclonal antibody for type VI, which was developed after the genetic cause was discovered), and antisclerostin (antibody to improve osteoblastic function) [52,54]. Bone tissue engineering plays an important role in the approach of antibody administration [55], as antibody therapies such as denosaub and antisclerostin are still undergoing clinical trials. More potential antibodies are undergoing research, such as the possibility of anti-TGFβ therapy to increase osteoclastic bone resorption [56]. In addition, many researchers are creating biologically inspired collagen and apatite composites to implant or replace and encourage bone regeneration [57]. Bone tissue engineering is also moving towards cell-based therapies, such as bone marrow [58] or mesenchymal stem cell (MSC) treatments [58,59,60]. Rather than preserving or increasing impaired collagen in the bone, the stem cells can be infused to differentiate it into osteoblasts and produce healthy collagen. A phase I/II clinical trial is currently underway to assess foetal safety and efficacy for MSC transplantation before and after birth to treat osteogenesis imperfecta [61,62].

EDS comprises a group of inherited connective tissue disorders affecting about 1 in 5000 individuals globally and is characterised by different degrees of skin hyperextensibility, tissue fragility, and joint hypermobility. Treatment is mostly supportive for symptoms, but a cure is not yet available as the molecular basis of EDS for hypermobility, the most frequent form of EDS, is still yet to be unravelled. Researchers are investigating the primary genetic defect and patho-mechanisms in order to make advances in providing effective therapy [63].

## 3. Sources of Collagen Type I

As has been documented since the early 1950s, collagen has traditionally been derived from bovine and porcine skins and bones. Collagen that is derived from fish, amphibians, and birds is also widely used. Another issue with porcine and chicken collagen is that it poses a high risk of disease transmission due to the outbreak of swine and avian flu [64]. Although there is little evidence that bovine collagen can contain bovine spongiform encephalopathy or mad cow disease and transmissible spongiform encephalopathies, it is advisable to be as careful as possible because of the severity of these diseases. Furthermore, porcine and bovine sources may be restricted due to religious reasons. As a result, alternative collagen sources are critical for providing potential biomaterials for biomedical and clinical applications.

Synthetic collagen, recombinant collagen, and gelatine are all popular ideas for clinical and biomedical applications. However, both synthetic and recombinant collagen have demoralised properties, and the high cost with low yields limits their use in tissue engineering. Synthetic collagen has a modified structure that differs from the natural microstructure and spatial complexity. Moreover, concerns with improper post-translational modifications, such as appropriate Pro and Lys hydroxylation, and biological functions that can affect the performance of recombinant collagen in clinical applications have yet to be resolved [4]. The gold standard for obtaining collagen type I is from mammalian species such as human xenogeneic donors. However, marine and fish collagen sources have also been thoroughly investigated and validated for future applications [65]. Another source of collagen is poultry by-products such as eggshell membrane, which demonstrated a low autoimmune and allergic reaction. Other sources of poultry collagen such as chicken bone, skin, and even duck feet have been successfully extracted with high yields [66,67,68,69]. Studies on alternative mammalian collagen type I sources include horse tendon [70] and ovine tendon [71].

## 4. Extraction of Collagen Type I

### 4.1. Salting-Out Method

The salting-out method was one of the first methods for extracting collagen. The basic idea is that collagen protein, and other proteins, is naturally soluble in salt. Therefore, neutral salt solutions such as sodium chloride (NaCl), tris(hydroxymethyl)aminomethane hydrochloride acid (Tris-HCl), and citrate can be used to extract soluble collagen proteins. The salt ions in solutions neutralise the surface charge of the collagen molecules, reducing electrostatic interactions between the collagen fibres and causing gradual precipitation. However, collagen in animal tissues is highly fibrous and crosslinked, which may restrict protein precipitation if the raw material has not yet been properly pre-treated.

An example of salt extraction procedures is based on tendon piece treatment with neutral salt solutions of 0.05 M disodium hydrogen phosphate (Na_2_ HPO_4_, pH = 8.7–9.1). The collagen is then separated by gradually increasing the NaCl concentration by adding 4 M of NaCl at 5 °C. To isolate the insoluble parts, the material can be precipitated one to four times and centrifuged. To avoid crystalline sodium phosphate formation at high salt concentrations, at the end of the process, potassium compounds such as potassium chloride (KCl) were used in later preparations, and the cation substitution did not affect the collagen properties [72]. The supernatant containing the salt-soluble fraction of collagen is then dialysed with 0.01 M Na_2_ HPO_4_ to produce a neutral solution [73].

This method has a high collagen recovery rate and does not affect the triple helix structure; therefore, it was a standard method in a previous study [74]. However, using this method, caution should be taken with the concentration of the salt as the key control factor. This is because salt such as NaCl may dissolve type I collagen in a neutral solution if less than 1 mol/L, but will precipitate type I collagen if more than 1 mol/L [75]. Furthermore, the removal of high concentrations of salt via dialysis may prolong the process, which typically takes three to seven days, thus reducing the extraction efficiency [76]. Moreover, mature collagen sources, such as adult animal tissues, are less soluble because most collagen molecules have already crosslinked over time. Only thin and fragile collagenous materials, such as defatted skin, are appropriate in this case. As a result, the salting-out method is the least desirable method for extracting collagen because it is unstable with limited use. However, salting-out is now commonly used in conjunction with acid extraction to recover protein after acidic hydrolysis, in which all non-collagenous substances are removed and crosslinks are cleaved, but collagen chains remain intact [77].

### 4.2. Acid Extraction Method

When compared to other methods, acid-based collagen extraction is the most commonly used because low acidic concentrations can disrupt ionic bonds and Schiff bases between the molecules. Organic acids such as acetic acid, lactic acid, and citric acid, as well as inorganic acids such as hydrochloric acid (HCl) can be used in acid hydrolysis. Organic acids, on the other hand, are more efficient [78] because they can solubilise non-crosslinked collagen, as well as break inter-strand crosslinks, resulting in a higher soluble yield [79].

An acid-based extraction method is usually performed by soaking the raw material, such as porcine or fish skin, in 0.5 M acetic acid at a controlled temperature until swelling (two- to three-times its initial volume) is visible between 24 and 72 h, followed by the precipitation process [80]. The precipitate is collected by centrifugation, and the supernatant is salted-out for 12 h with NaCl. The supernatant is redissolved in 0.5 M of acetic acid and dialysed for a few days against cold distilled water with a molecular weight of 7 kDa before being lyophilised and stored at −20 °C until further use [81].

Through non-selective chemical hydrolysis of collagen chains, the acid degrades the various amino acids in the sample and cleaves non-covalent intermolecular and intramolecular bonds [82]. This method is better suited for fragile materials with fewer intertwined collagen fibres such as porcine and fish skins. However, collagen extraction from large mammalian tendons, such as ovine tendon, has also been successful [71,83].

### 4.3. Alkali Extraction Method

The alkali method is based on chemical hydrolysis as well; however, it employs a basic solution, typically sodium hydroxide (NaOH), and the process can take several days to several weeks [80]. This method is better suited for thick materials because it penetrates more aggressively, such as leather waste, for example bovine shavings, to swell the material and break down non-collagenous substances [82]. In some cases, alkali is suitable for thin materials for faster processing [79]. Hattori et al. used alkali-based extraction for 2 weeks at 20 °C, soaking in 3 percent NaOH (*w*/*v*) and monomethylamine, and the resultant collagen was precipitated by dialysis in 50 mM Tris-HCl buffer (pH 7.4). The collected precipitate was dissolved in 5 mM acetic acid [84].

However, this method is less advantageous compared to acidic extraction because different concentrations of the base can result in the loss of soluble collagen and structural modifications. For example, Liu et al. used NaOH to hydrolyse grass carp skin, a type of fish, and discovered that NaOH at concentrations ranging from 0.05 to 0.1 M was effective in removing non-collagenous proteins, but soluble collagen was lost at 0.2 M and 0.5 M, while structural modification in the collagen was observed at 0.5 M [79].

Although the alkali method has a high recovery of collagen and can maintain its property as a biological adherent molecule to human keratinocytes and fibroblasts, alkali-treated collagen loses its ability to form fibrils at neutral pH under physiological conditions when compared to acid-soluble collagen [84]. The alkali method produces a high yield of collagen protein, but all amino acids containing hydroxyl and sulfhydryl may be destroyed [82]. However, high-quality collagen can still be extracted using this method by optimising a few variables such as the temperature, concentration, pH, and process time.

### 4.4. Enzymatic Extraction Method

Enzymes can also be used to break down crosslinked bonds and produce biologically active collagen with intact amino acids. Pepsin, tryptase, and papain [82] are the most commonly used enzymes for collagen extraction, as are commercially available enzymes such as alcalase and flavourzyme [80]. The enzymes break down the crosslinking in the collagen by cleaving amino telopeptides from the tropocollagen molecule, thus allowing it to be dissolved. This is most useful in mature tissue, whereby strong intermolecular bonds have formed as a result of ketoimine crosslinks [75]. Enzymatic extraction is frequently combined with acid to increase the yield and shorten the reaction time.

Enzymatic hydrolysis begins with the addition of the raw material, or residue from acidic extraction, to an acid such as 0.5 M acetic acid or 0.01 M HCl containing selected enzymes such as 0.1 percent (*w*/*v*) pepsin. The mixture is continuously stirred at 4 °C for approximately 48 h. The solution is then filtered or purified, and the supernatant is salted-out to precipitate. The resulting solution is centrifuged, followed by acid redissolution, dialysed to obtain acid-soluble collagen, and lyophilised [73,85]. Wang et al. extracted collagen from Amur sturgeon skin using three methods, namely salting-out, acid extraction, and the enzymatic method, and discovered that while all three had comparable amine contents, their yields were vastly different [85]. Salting-out with NaCl provided the lowest yield of 4.55%; acid-soluble collagen yielded 37.42%; the pepsin enzymatic method provided the highest yield at 52.80% [85].

Enzymatic hydrolysis is more specific in cleaving specific functional groups in the collagen chain, thus allowing control over the degree of hydrolysis. Therefore, it is less destructive to the collagen protein and has a lower overall salt content. However, enzymes are more expensive, but this method may be justified because it produces a higher yield of collagen polypeptides. Depending on the type of enzyme used, the enzyme method may require a longer reaction time and optimal designs in pH, temperature, and time.

### 4.5. Ultrasound Extraction Method

To improve mass transfer in liquids, ultrasound is widely used to improve processes requiring mixing, dispersing, and extraction. Ultrasonication is a technology that uses high-frequency (20 kHz) sound waves to create a phenomenon known as cavitation. Cavitation bubbles collapse so quickly that there is massive turbulence, resulting in extreme pressures and temperatures [86]. Petcharat et al. demonstrated that ultrasonication at an 80% amplitude for 10 min increased collagen yield from clown featherback fish without affecting collagen’s molecular structure. Furthermore, case studies by Zou et al. [87] and Akram and Zhang [88] compared the extraction of chicken collagen with and without ultrasonication, as well as with and without pepsin and discovered that ultrasound power significantly increased the extraction rate and equilibrium concentration. Both studies reported that FTIR results showed intact collagen triple helical structures. In addition, ultrasound-treated collagen with pepsin enzymatic hydrolysis resulted in superior thermal stability and functional properties, which include solubility and the ability to form fibrils. Ultrasound is an alternative and faster method of extraction, which is gaining popularity. However, appropriate conditions are required because harsh conditions, especially for long periods, will affect the material structure and characteristics. Rapid changes in temperature, pressure, and strength can break the van der Waals forces and hydrogen bonds in the collagen chains, resulting in denaturation and impure collagen polypeptides [89].

### 4.6. Collagen Extraction Process

**Figure 3:**
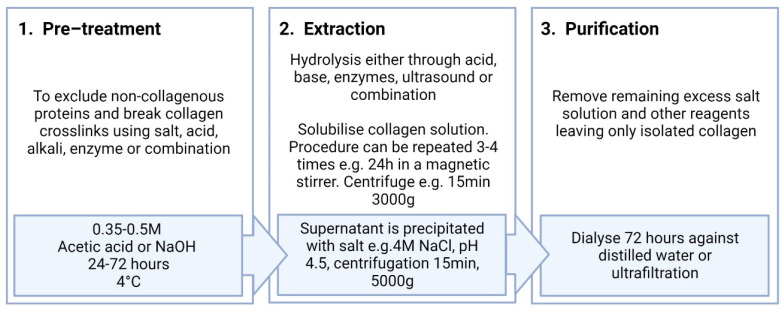
Steps of the collagen isolation process: an overview. Examples of methods from Mogan et al. [73]. Different tissues require different optimisation in the reagent’s concentration, pH, processing time, and temperature.

The collagen extraction process can be divided into three steps using the methods described above, namely (1) pre-treatment, (2) extraction, and (3) purification. Figure 3 depicts a high-level overview of the processes. Collagen must be pre-treated, which may involve the use of alkali or acid, depending on the source of the material. To obtain higher pure collagen yields, pre-treatment is required to break down the non-collagenous substances and crosslinks. When raw materials are immersed in an acidic or base solution for an extended period, the solution is absorbed by the material, causing it to swell significantly and disrupt non-covalent intermolecular and intramolecular bonds [80].

Following that, extraction is accomplished through chemical hydrolysis using an acid or base method, enzymatic hydrolysis, or ultrasound. The goal of partial hydrolysis is to break the crosslinks of collagen fibres while leaving the long polypeptide collagen chains intact. The method’s application is determined by the solubility of collagen in acid solutions, neutral saline solutions, alkali solutions, and acid solutions containing enzymes. Several covalent intramolecular and intermolecular crosslinks, primarily those involving protein and polysaccharide bonds such as lysine and hydroxylysine residues, as well as bonds with saccharides and esters, must be discarded [90]. After extraction, the material is precipitated with salts to make the collagen less soluble and separate from the liquid residue. To remove any remaining salt residues, the precipitate can be collected, resolubilised, filtered, or dialysed after centrifugation. Table 1 provides a summary of collagen type I sources, extraction methods, and scaffold comparisons. In general, acid or an enzyme such as pepsin is preferred for extraction, salt or neutral solution for precipitation, and bases for quick pre-treatment.

## 5. Physicochemical Characterisation of Collagen Type I

Collagen type I has specific characteristics in its molecular weight, biocompatibility and immunogenicity, three-dimensional stability (mechanical and thermal strength, porosity, and biodegradation), swelling ratio, water permeability, and surface and chemical structure. Each of these can be tested using various methods described in more detail below. The parameters in measuring the various physicochemical characterisations allow investigators to select the best biomaterial development in the formulation and selected fabrication methods. Table 2 provides a summary of the different physical and chemical characteristics exhibited by collagen type I, as well as the various methods and expected results for each method.

### 5.1. Parameters and Test Methodologies for Biomaterial Analysis

Material characterisation is crucial as it is the first step of the biological evaluation process. The extent of the chemical characterisation needed is dependent on the existing safety and toxicological data of both pre-clinical and clinical data and the nature and duration of the contact of the biomaterial with the body. At the very least, characterisation addresses the constituent chemicals of the device, as well as possible residuals used. The various methodologies to measure collagen type I are based on biomaterial physico-chemical, morphological, and topographical (PMT) characterisation, as well as chemical and biocompatibility as general parameters that many bioengineers rely on. The international standards to evaluate the PMT and chemical characterisation of medical devices are ISO 10993-18 and 10993-19 as general guides. Table 2 shows investigations performed specifically on type I collagen. However, the expected results from Table 2 are according to collagen type I, and they may differ based on the sources of collagen, the extraction method, and the formulation of biomaterial, particularly if augmentations were included, such as additional biochemical factors such as nanoparticles. For example, after the initial test of a cell–bioscaffold interaction for biocompatibility, material characterisation for biological assessment for medical devices may also consider chronic toxicity, carcinogenicity, sensitisation, genotoxicity, implantation, and hemocompatibility (ISO 10993-1:2009). Table 2 focuses on the in vitro initial techniques that can be performed to select the biomaterial formulation during fabrication.

That said, researchers may have other alternatives or improved techniques that can be utilised as technology advances. For example, a study in 2019 discovered the lattice arrangement of human dermal collagen and elastin using a combination of multiphoton imaging and biaxial tissue extension [34]. Alternative methods are also based on the investigator’s application and resources. For example, thermal stability can be measured using many instruments including differential scanning calorimetry (DSC), thermogravimetry analysis (TGA), thermal mechanical analysis (TMA), or dynamic mechanical thermal analysis (DMTA), which measures both mechanical and thermal stabilities.

**Figure 4:**
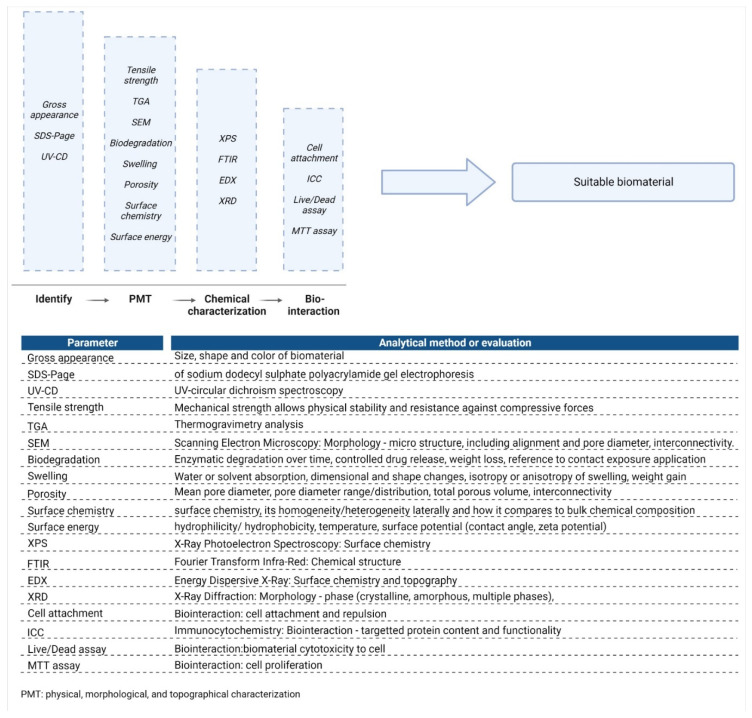
Parametric selection for the most suitable biomaterial.

Nevertheless, principally, the consideration of the in vitro characterisation for materials in medical devices is a necessary first step to assess the biological safety and biomimetic capability of the device. It is also important to judge whether the proposed fabricated material is comparable to the characteristics of established material since type I collagen is extensively researched with clinical significance. As such, the parameters described could be used as an initial selection process regarding the biomaterial’s suitability (Figure 4). More discussion on the parameters for each characterisation is given in their respective subsections below.

### 5.2. Characterisation of Purified Collagen Type I

The total yield of collagen extraction is determined by several important factors, namely the source species, tissue types, extraction methods, and other factors that protect the collagen content from degradation. Collagen content can be measured using various methods such as lyophilisation and weighing [93] or through specific testing such as the Sircol assay [71] and hydroxyproline analysis [125]. By using an acid-based extraction, the collagen content extracted from rat tail was found to provide a higher yield compared to bovine tendon [93]. Furthermore, it was discovered that porcine collagen yielded more collagen than avian collagen [106]. To date, the yield of collagen content for various species, such as mammalian, fish, amphibian, avian, and marine, is significantly different.

Collagen purity is commonly determined using the traditional technique of sodium dodecyl sulphate polyacrylamide gel electrophoresis (SDS-PAGE). It is critical to validate the purified collagen after it has gone through all of the purification procedures. On SDS-PAGE, three bands are commonly found for collagen type I, which refer to the basic structure of monomeric alpha-1 and alpha-2 monomers, as well as the common motif of regular collagen secondary structures of the β-band and γ-band. The alpha bands of type I collagen are approximately 100–130 kDa, while the beta band is approximately 250 kDa [107]. The β-band represents a dimer representing two alpha chains crosslinking with each other, and the γ-band represents three alpha chains, which are visible in the upper part of the gel [126].

It is also critical to determine whether the isolated collagen type I has a native triple helical structure or a denatured coil structure. UV-circular dichroism (CD) spectroscopy can represent collagen’s polyproline-II-like triple helical shape by a positive maximum absorption band at around 222 nm. In a study comparing collagen solutions from codfish, bovine skin, and rat tail, a significant band was found for bovine, less so for rat tail, and ambiguous for codfish [108]. If there is no positive band at 222 nm, partial denaturation can be assumed. Diluted acids and repulsion forces between identical charges of collagen monomers dissociate the triple helix’s intermolecular connections. On the other hand, intramolecular interactions are determined by the amino acid composition, specifically the presence of proline and hydroxyproline, which provide stereochemistry with the pyrrolidine ring and may form additional hydrogen bonds.

### 5.3. Biocompatibility and Immunogenicity Properties of Collagen Type I

When a cell and a material come into contact, information is transferred from the surface of the material to the cell, and the interaction may cause both components to change. Biomaterials must be biocompatible and free from immune response to be developed as a functional bioscaffold. Biocompatibility is defined as the ability of the biomaterial to perform its function without causing any local or systemic effects [127]. To date, the international standard ISO 10993: Biological Evaluation of Medical Devices has been used to standardise the biocompatibility evaluation of a medical device. Under ISO 10993-Part 5: Tests for in vitro cytotoxicity, any material could be tested either by extraction or direct or indirect contact interventions. For biocompatibility testing of collagen extracts, the 3-(4,5-dimethylthiazol-2-yl)-2,5-diphenyltetrazolium bromide (MTT) assay is commonly used compared to the live and dead assay, apoptosis assay, or annexin V assay [99]. Besides that, the SEM of treated cells at higher magnification can provide scientific evidence about cellular compatibility by observing the morphology of the cells on the biomaterial. Other proliferation assays can also be used such as the cell counting kit-8 (CCK-8) because it is non-toxic to the cells and reusable, which is important for difficult-to-culture cells. Apart from that, the AlamarBlue or PrestoBlue proliferation assays could also be used. Immunogenicity testing is also required for the evaluation of a biological device under this ISO guideline. Immunogenicity refers to the ability of the substance or material to elicit an immune response in the body system. Immunogenicity testing is essential, and it is also a regulatory requirement for any substances or materials that will be used for clinical applications or therapy.

Furthermore, the intrinsic binding sites of the collagen are critical for biocompatibility and should be intact after isolation. Adhesion proteins (such as fibronectin, collagen, laminin, and vitronectin) and specialised cell receptors (integrins) that are attached to the cell membrane allow cells to adhere to the surfaces. When fibroblasts grow on a substrate, the majority of their cell surface is separated from the substrate by a gap of more than 50 nm. However, this gap is reduced to 10 to 15 nm at the focal contacts. The integrin family contains the primary transmembrane linker proteins of focal contacts, and the cytoplasmic domain of the integrin links to talin, which then binds to vinculin, a protein found in other actin-containing cell junctions. Vinculin binds to α-actinin and is thus associated with an actin filament [128]. The cell attachment assay and immunocytochemistry (ICC) on integrin-related proteins such as phalloidin and vinculin can verify that the biomaterial from the isolated collagen has intact integrin binding sites. An ICC for Ki67, for example, can be used to validate the data of cell proliferation on the bioscaffold, as it is a direct and distinctive cell proliferation marker compared to the indirect marker of colour-based assays such as MTT.

The current findings from type I collagen in vitro and in vivo model studies have not accurately represented the nature of the immune response in the human body. However, the outputs at least enabled early determination of an acute or chronic immune response post-implantation. In in vivo experiments, tissue response to type I collagen nanostructured bioscaffolds has previously formed protein corona, a macromolecular coating complex on the surface of transplanted nanoparticles when in contact with the physiological environment such as blood [129]. The physiological environment includes blood, interstitial fluid, and cellular cytoplasm containing complex protein mixtures such as albumin, fibrinogen, fibronectin, gammaglobulins, and vitronectin and other molecules such as lipids, sugars, and ions [130]. When engineered proteins interact with such physiological environments, they automatically adsorb the protein mixtures around them on the implant surface, called the “Vroman effect” [131]. This response is also affected by the biomaterial surface characteristics such as energy, chemistry, roughness, and topography [132], which makes these parameters important in bioscaffold design.

The formation of corona due to the Vroman effect was reported to influence cell–biomaterial interactions, whereby corona complexes formed on fibrils of a bioscaffold have distinct and reproducible compositions that modulate tissue microenvironment and can indicate the subject’s health condition [133]. The tissue responds to foreign bodies, such as the engineered bioscaffold, by having this molecular complex on the surface that directs adhesion and cross-talk between macrophages on the foreign body and inflammatory or wound healing cells [134]. As implants remain in the tissue for an extended time, fibrous encapsulation can form and bring complications to the implantation. The formation of scar tissue on and around implant materials hinders the ability of the material to function and can call for extraction surgery. Stiff capsular contraction can also cause chronic pain and limit the mobility of the patient. However, this can be prevented by designing biomaterials with specific properties [135]. Porous and biocompatible bioscaffolds will not form fibrous capsules because connective tissue can grow within the pores and interact favourably by promoting native tissue adhesion and vascularisation since molecules can pass through and interact. Biodegradable scaffolds also prevent fibrotic encapsulation because they are eventually metabolised by the body and removed. Biomaterials that limit myofibroblast formation can also reduce the formation of stiff scar tissue [136]. An ICC on the alpha-smooth muscle actin of cells on the bioscaffold can indicate myofibroblast formation.

ISO 10993-Part 5 also provides guidance on the biological assessment of biomaterials including the irritation and sensitisation test. Both tests were developed to provide beneficial scientific findings on the skin and mucous membrane irritation and skin sensitisation potential. These tests do not provide a clue to any other adverse effects besides the aforementioned effects. Therefore, several other tests to validate immune responses have been introduced, which include screening assays, confirmatory assays, and comparative studies (under WHO guidelines) involving clinical trials among the most sensitive patient populations [137].

### 5.4. Three-Dimensional Stability

Mechanical strength, thermal stability, porosity, and biodegradation influence collagen type I stability. It is critical to create a bioscaffold that is sufficiently stable and does not interfere with biological functions [138]. Furthermore, structural stability aids in cueing cell behaviour within the three-dimensional structure.

#### 5.4.1. Mechanical Strength

When developing collagen-based bioscaffolds, since there is no gold standard for tensile strength, it depends on the multiple formulations, characteristics, and state of the polymer sample. However, because tensile strength varies greatly between tissue types, it should resemble the strength of the targeted native tissue. Generally, the mechanical strength of collagen type I in fish and aquatic sources is lower than in mammalian, avian, and other higher orders of families [110]. However, there are exceptions in large marine animals, such as the giant squid mantle, which have better collagen mechanical characteristics than those of humans [139].

**Figure 5:**
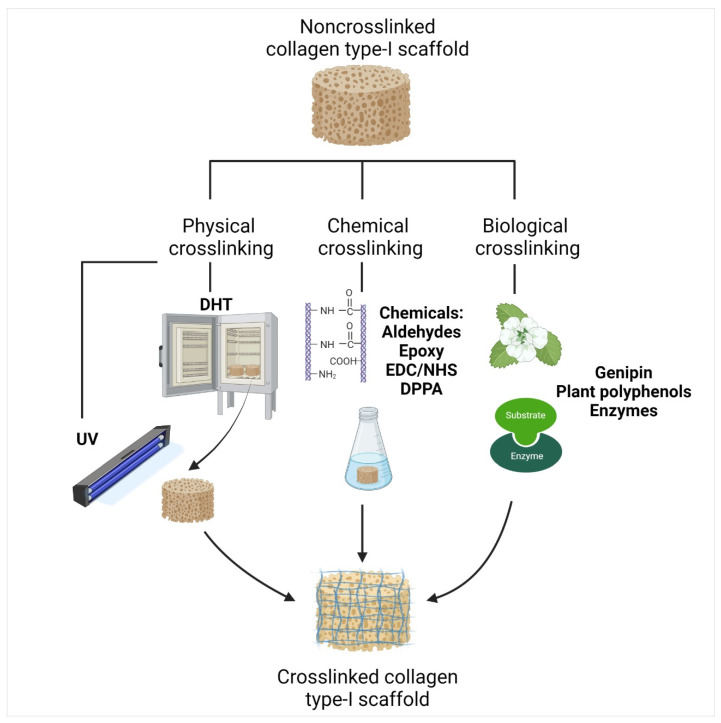
Illustration of different techniques for crosslinking collagen type I scaffolds. Physical crosslinking includes physical alterations, usually from DHT heat treatment or UV irradiation. Chemical crosslinking refers to chemical alterations using aldehydes such as glutaraldehyde, epoxy compounds, commonly used EDC/NHS crosslinking, and DPPA. Biological crosslinking uses materials derived from biological sources such as genipin, a plant-based crosslinker, plant polyphenols, and enzymes.

Mechanical strength is influenced by a variety of factors, including three-dimensional scaffold design [140] and crosslinking intervention [71,109]. Collagen type I is particularly soft and fragile when left alone. By itself, its mechanical stability and mechanical integrity are inadequate for tissue regeneration applications. Mechanical enhancement is critical for maintaining physical stability, thus allowing it to be used for tissue regeneration [141]. It is possible to achieve this through crosslinking via physical, chemical, biological, or combination intervention methods. In developing collagen-based biomaterials, crosslinking enhances the chemical and physical interactions between polymers in the structure, thus forming a denser matrix network. This makes them more stable, have controllable biodegradable rates, improves biological properties such as reducing cell-mediated contraction, and have increased load-bearing performance from the stronger tensile strength [142]. Crosslinking is defined as the process of joining two or more molecules by a covalent bond, intermolecularly or intramolecularly, or both. The categories of crosslinkers and their examples are illustrated in Figure 5.

**Scheme 1:**
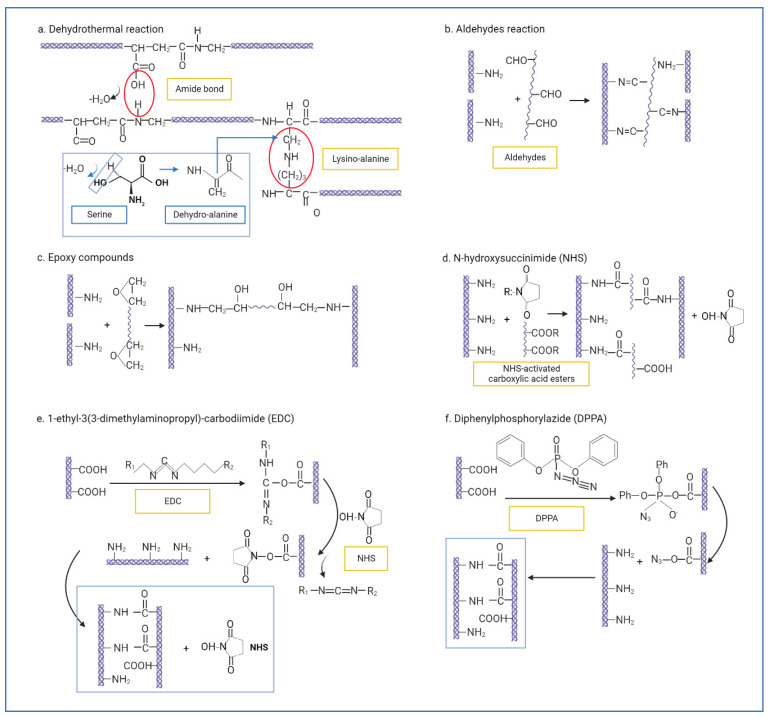
Schematic illustration of crosslinking mechanisms of type I collagen with a physical crosslinker, (**a**) dehydrothermal treatment (DHT), and chemical crosslinkers, (**b**) aldehydes, (**c**) epoxy compounds, (**d**) NHS-activated carboxylic esters, (**e**), EDC and EDC/NHS, and (**f**) DPPA. These figures were adapted from Zhang et al. 2020 [112].

Physical crosslinks involve physical alterations on the scaffold, such as dehydrothermal treatment (DHT), ultraviolet irradiation (UV), or other forms of irradiation such as gamma rays and electron beams. DHT involves heat treatment of dry collagen in a vacuum oven at 100–140 °C for 24 to 72 h or more [143]. During DHT, amide bonds are formed in the collagen structure between amine and carboxyl groups, and lysino-alanine bonds are formed between dehydro-alanine and lysine residues. These chemical reactions take place due to the heat, thus causing intramolecular and intermolecular crosslinks (Scheme 1a) [144]. It was found that DHT can increase the crosslinking density with increasing temperature, and this will increase the tensile strength of the collagen film up to three times compared to the untreated collagen film [145]. Thermal degradation occurs simultaneously with crosslinking during DHT treatment. Therefore, parameters for treatment should be adjusted depending on the collagen sources and the formulation of the biomaterial. UV, gamma ray, and electron beams form intermolecular links by generating free radicals [146]. Irradiation treatments are often used for sterilisation and together with other forms of crosslinking, because although UV was found to be non-disruptive to cells, by itself, it is not enough to produce a high crosslinking density [142].

Chemical crosslinking refers to using chemical reagents to crosslink native collagen or collagen biomaterials and is carried out by introducing functional groups into collagen molecules. Examples include using aldehydes such as glutaraldehyde (GA) or dialdehyde starch. Epoxy compounds and N-hydroxysuccinimide (NHS) react directly with free amino groups in between different collagen chains [147]. Crosslinking by using 1-ethyl-3(3-dimethylaminopropyl)-carbodiimide (EDC) and diphenylphosphorylazide (DPPA) starts by activating carboxyl groups within collagen chains and promotes amido bond formation between adjacent collagen carboxyl and amine groups [148]. Normally, EDC/NHS crosslinking is combined to speed up the process as they complement each other in activating carboxyl groups and promote crosslinking interaction [112].

Previously, GA was used to crosslink collagen biomaterial, but it is often cytotoxic. A study was performed to compare different collagen membranes crosslinked with DPPA and GA cultured on human fibroblasts. Membrane crosslinked with GA had reduced cell growth and collagen secretion compared to DPPA. A collagen sponge (bovine) was fabricated and crosslinked with DPPA seeded with foetal bovine epiphyseal chondrocytes, showing positive cell proliferation over 4 weeks in culture without biodegradation [149]. DPPA-crosslinked collagen sponges seeded with allogenic rabbit chondrocytes can repair partial thickness cartilage defects in the rabbit knee. Although DPPA offers quick and effective results, it is often not possible to completely remove the solvents [150], which may have adverse effects if there are excessive chemicals in vivo.

A phase I clinical study was conducted with a similar construct for corneal implants, but it was made from recombinant human collagen (RHC) hydrogels crosslinked with EDC [151]. Two years after surgery, all 10 patients had regenerated their epithelium, stroma, and nerves and restored tear film and touch sensitivity in their corneas. After three years, the corneas were stable without immunosuppression. However, early implants had microcracks and shearing, which made sutures necessary, but the tight suturing impeded epithelial growth, resulting in astigmatism, haze, and graft thinning. In this case, EDC crosslinking or formulation was not sufficient nor too rigid for the application and required alternatives for more elasticity.

Therefore, the epoxy compound was used to crosslink collagen (from porcine) and collagen-laminin peptide hydrogels as corneal substitutes due to their ability to increase mechanical strength and maintain some pliability [152]. A bifunctional epoxy-based crosslinker, 1,4-butanediol diglycidyl ether (BDDGE), was used and showed biocompatibility with corneal epithelial cells and the ability to be tailored to the needs as the mechanical properties could be modified based on the state in which the hydrogels were fabricated. Hydrogels that were crosslinked with BDDGE and EDC/NHS resulted in higher elasticity. In addition, slow gelation time opens the opportunity for drug encapsulation, and the addition of laminin peptides of YIGSR and IKVAV can enhance the growth of epithelial and nerve cells. Since it is pH dependent, optimising the parameters of the pH, collagen concentration, and combination or follow-on crosslinkers is necessary. Kozlowska et al. fabricated a scaffold and compared the crosslinking effects between DHT and EDC/NHS [153]. The material used was collagen isolated from the tendons of young rats combined with calcium phosphate as a potential for bone tissue engineering. It was found that although both have similar mechanical strength post-crosslink, DHT is much more susceptible to enzymatic weight loss up to 69.6% and has a much slower absorption rate. However, it has been reported that EDC/NHS has a poor modulation of foreign body response after implantation [154]. The structural formulas for each of the crosslinking mechanisms for the physical and chemical crosslinking method are depicted in Scheme 1.

**Scheme 2:**
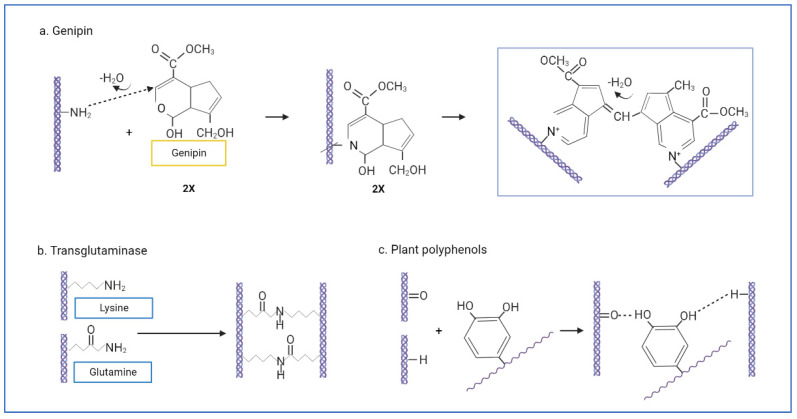
Biological crosslinking mechanisms of type I collagen for (**a**) genipin, (**b**) transglutaminase, and (**c**) plant polyphenols [112].

Biological crosslinkers refer to using crosslinkers derived from biological sources to overcome some shortcomings of physical crosslinkers and possibly toxic chemical crosslinking (Scheme 2). Some identified biological crosslinkers include genipin, transglutaminases, and plant polyphenols (variations include procyanidin/proanthocyanidins). Genipin is a hydrolysed product of genipin glycoside extracted from the fruits of Gardenia jasminoides [155]. Their crosslinking reaction is achieved in two phases: (1) the C3 carbon of the genipin ring reacts with the primary amine group within collagen, creating an aldehyde group that reacts with a secondary amine group to form a heterocyclic compound by removing oxygen in the process; (2) two of the heterocyclic compounds link together via ester group substitution by a secondary amide bond (Scheme 2a) [156]. Genipin has low toxicity, almost 5–10,000 less toxic compared to GA [157]. Transglutaminase (TG) is an isolated microbial enzyme that catalyses acyl transfer reactions predominantly involving glutamine and lysine residues of collagen (Scheme 2b) [158]. TG takes part in blood coagulation, regulation of red blood cell membranes, and epidermal keratinisation [159]. Crosslinking scaffolds with TG improves chondrocyte viability in vitro and in vivo [160,161]. However, enzyme deregulation may cause harmful problems [158]. Plant polyphenols (PA), also called vegetable tannins, modify collagen by multipoint hydrogen bonds formed between the phenolic hydroxyl groups of polyphenols and the hydroxyl, carboxyl, amino, or amide groups of collagen chains (Scheme 2c) [162]. A fibroblast cytotoxic assay revealed that PA is approximately 120-times less toxic compared to GA. The PA crosslinked biomaterial has enhanced collagen expression and deposition, as well as no calcification after implantation in bone tissue engineering [162]. Proanthocyanidin (PC) is highly attractive due to its antioxidant, anticancer, antimicrobial, antiangiogenic, and anti-inflammatory properties [163]. However, their applications are limited because of their high sensitivity to light, thermal exposure, oxidation, and even poor absorption in the gastrointestinal tract. As such, investigators try novel approaches such as microspheres to improve PC stability and bioavailability for drug delivery [163]. More biological crosslinkers are being studied, including quercetin, epigallocatechin-3-gallate (ECGC) from green tea polyphenol, tyrosinase, lysyl oxidase, phosphatases, and horseradish peroxidases (HRPs), which can be further explored to formulate the optimal collagen biomaterial.

Table 3 provides the classification of the crosslinkers and the detailed mechanism of action and highlights the benefits or disadvantages of each crosslinking method described. The schematic illustration of structural chemical formulas for the different crosslinking methods for collagen type I are depicted in Scheme 1 and Scheme 2.

#### 5.4.2. Thermal Stability: Denaturing Temperature

The denaturing temperature (T_d_) of collagen type I refers to the transformation of the native form to the denatured form, in which the hydrogen bonds are broken and α-chains dissociate, which is often irreversible. When enough heat is applied, the structural H-bonded water content integral to the structure of the molecule is released [171]. This varies depending on the source (species and tissue type) and correlates with the levels of proline (pro) and hydroxyproline (hyp) content, which determine the 3D stability [172]. For example, the T_d_ of collagen from diamondback squid is 27.5 °C, which is 10 °C lower than porcine collagen [173]. The hyp content (residues/1000 total residues) in diamondback squid is 89 [173], which is also lower than the 97 in porcine collagen [174]. However, even within the same species, the thermal stability of collagen differs based on distinct living environments. For instance, annelids living in colder temperatures have lower T_d_ and hyp content compared to those living in shallow waters with warmer temperatures [175].

The denaturing temperature is proportional to the thermal stability, indicating that a higher T_d_ means a more thermally stable structure. Zhang et al. [112] made excellent detailed comparisons on how other factors such as extraction methods, different sources in species and tissue types, solvent systems, polymer design, and crosslinking may affect the thermal stability of collagen. In general, the T_d_ of collagen extracted from warm-blooded terrestrial mammals ranges between 37 and 40 °C; aquatic animals possess a lower T_d_ range of 26–37 °C; colder marine life such as deep-sea animals have a T_d_ range of 6–20 °C [111,112,113]. The denaturation temperature of collagen molecules is typically the body temperature because type I collagen is thermally stable below body temperature [176]. Contrary to the widely held belief, nature’s preferred conformation of collagen in physiological solution is random coils instead of the triple helix, and the hyp content is able to be adjusted to maintain equilibrium [176]. However, the thermal stability in fibres, films, and sponges is higher than collagen in solution due to the intramolecular and intermolecular interactions in higher-ordered collagen structures. While collagen triple helices are marginally less stable in physiological conditions, the T_d_ of collagen fibres is approximately 60 °C at normal water content. The T_d_ of native hydrated collagen fibrils from rat tail tendons is 65 °C [114], and the stabilisation temperature of collagen fibrils ranges from 58–75 °C [177].

Although it is generally accepted that most collagen extracted from aquatic sources exhibits lower thermal stability compared to mammalian collagen [178], there are some exceptions to this rule for certain aquatic species [179]. Furthermore, T_d_ can be significantly improved by crosslinking [113]. The need for greater thermal stability is critical in the development of biomaterials for regenerative medicine and biomedical applications [180]. Ideally, the thermal stability of biomaterials should be similar to or higher than that of native tissue without compromising other physico-chemical characteristics. For instance, the T_d_ for collagen in native human skin is in the range of 57–65 °C [114]. Two blends of ovine collagen–gelatine–elastin (CollaGee) biomaterial were fabricated as a skin substitute, and it was found that, without crosslinking, the T_d_ were 64.5–69.12 °C and 57.37–61.29 °C, respectively [181]. Although this is within the range of the native skin T_d_, the biomaterial biodegradation was not satisfactory, and upon crosslinking with genipin, the T_d_ increased to 76.89–81.70 °C and 92.50–99.75 °C, respectively. A fish collagen scaffold from silver carp had an increased T_d_ of 77 °C to 80 °C after DHT crosslinking and 88 °C after crosslinking with EDC/NHS modifications [180]. It is most likely that fabricated bioscaffolds from different sources of type I collagen can have higher thermal stability than native tissue by crosslinking for tissue engineering applications.

#### 5.4.3. Porosity and Pore Size

To improve cell survival and fate within the layer structure, a 3D scaffold with appropriate pore sizes and porosity is required. This environment should be similar to the extracellular matrix of the tissue, which varies significantly depending on the tissue type. It would allow cells to respond to mechanical cues provided by the designated bioscaffold by behaving and interacting [182]. In general, a smart bioscaffold should be a porous structure with highly interconnected pores that allows nutrient and oxygen diffusion, biological waste removal [183], and a 3D environment in the scaffold for cell assembly and differentiation [184]. Furthermore, by providing sufficient space in the scaffold, interconnected pores promote cell seeding, growth, proliferation, and tissue formation.

In developing biomaterials, the mean pore diameter, pore range and distribution, total porous volume, and interconnectivity are considered. The methods for this parameter can be by SEM, water vapour transmission rate (WVTR), and liquid dispersion. The acquired data should be relevant to the bioscaffold application. For instance, more than 90% porosity is required to support osteoprogenitor cells, which is an ideal scaffold for bone [185]. Meanwhile, to increase the interconnection between the surface and the inner bulk, particles of ice can be embossed on the surface of the scaffold. Because of the compact packing of the pores, this method has been scientifically proven to increase mechanical strength by >15-fold over other available methods. A well-connected pore structure, according to this statement, ensures a homogeneous cell distribution on the scaffold [186]. The pore size in the range of 150–250 µm is ideal for cell distribution and smoothing cell delivery onto the scaffold [184]. Additionally, porous scaffolds can serve as vital delivery system carriers. The interconnected pores of the porous scaffolds increase their capacity for transporting and loading, and many of them can deliver with relatively big dimensions, such as living cells and proteins [187].

The optimal pore size varies depending on the type of cell. This is because pores larger than 80 µm are ideal for fibrogenic ingrowth, whereas pores smaller than 20 µm are required for chondrogenic growth. Meanwhile, pore size varies depending on the derivative. The average mean size for ovine-, bovine-, and porcine-derived scaffolds in this scenario is 73.05 ± 10.79 µm, 85.84 ± 9.51 µm, and 87.32 ± 10.69 µm, respectively [115]. To date, researchers have used different techniques to create collagen scaffolds with varying porosity, including freeze-drying, phase separation, salt-leaching, 3D printing systems [182,188], gas-forming foam, treatment using laser, electrospinning, and crystal precipitation [185]. On the contrary, naturally derived polymers have a significant impact on the porous scaffold. This is because natural polymers contribute to low mechanical strength [186]. On the other hand, building a living tissue substitute that is functionally, mechanically, and structurally similar to native tissue remains a challenge.

#### 5.4.4. Biodegradation

Biodegradation of the collagen scaffold must occur at a proper time to allow for cell invasion and blood vessel growth in the collagen matrix, which stimulates the formation of new tissue. The body may respond with an inflammatory reaction as a result of the implant material or the material may release metal ions or treatment drugs based on its application. Proinflammatory cytokines such as interleukin-6 (IL-6) and tumour necrosis factor alpha (TNF-α) can produce substantial acute-phase proteins, which drive the biodegradation process [189]. The granulocyte and monocyte or macrophage systems are stimulated, causing them to migrate to the site of inflammation (chemotaxis) and supplanting them with the adhesive and opsonising properties of bacterial antigen proteins to promote biodegradation. It is critical to design a bioscaffold that can withstand the body’s initial response so that it does not degrade immediately, but instead encourages cell signalling for regeneration over time.

Rapid biodegradation may also be caused by the porous structure, which is a disadvantage for native collagen [190]. Chemical, physical, and enzymatic crosslinking can be used to control collagen scaffold biodegradation based on the current demand. The biodegradation property of collagen type I improves tissue structure and functionality by stimulating fibroblasts in humans. Thus, the time required for re-epithelialization is greatly reduced [191]. Collagenase (MMP-1) will disrupt the triple helix structure of collagen type I in human tissue, thereby hydrolysing it. This will produce collagen fragments. This is then degraded by other proteases, such as gelatinases. As a result, no foreign residues are left during the degradation process because biodegradation in the human body is regulated by the action of metalloproteinase enzymes [192].

Biodegradation is also proportional to mechanical strength, as collagen structures with fewer crosslinks will be much more susceptible to enzyme degradation. Furthermore, scaffolds with larger pores have more stretched-out collagen fibres, which is why shrinkage post-crosslinking is also measured to reduce porosity in a way that maintains scaffold ability for absorption and cell migration without compromising the structure of the scaffold. The parameters of mechanical strength, thermal stability, porosity, and biodegradation that we have described so far are characteristics of the collagen scaffold that relate to each other. Hence, this provides guidance to formulate the biomaterial.

There is no gold standard for the biodegradation rate as it depends on the state and design of the biomaterial. However, wound dressings were changed after 7 days in an in vivo model by Mh Busra et al. [116]. Given that a normal deep wound in healthy patients can take up to 14 days to heal, a good biomaterial should be able to degrade in 3–7 days for rapid wound healing.

### 5.5. Swelling Ratio

Swelling affects not only the efficiency of the scaffold, but also its water adsorption capacity. The degree of crosslinking, the presence of hydrophilic groups, pore size, and pore interconnection influence the swelling ratio [193]. At the same time, the swelling capacity influences the enzymatic degradation of a scaffold. The swelling ratio of pure collagen is approximately 650% due to the presence of hydrophilic groups [194]. However, the swelling ratio varies depending on the source of the collagen derivative. Ovine collagen, for example, is twice as resistant to collagenase degradation as bovine and porcine collagen. The swelling ratios of collagen type I from ovine, bovine, and porcine sources are estimated to be 2500%, 2750%, and 2700%, respectively [115]. In tissue engineering, the swelling ratio favours the lesion exudate environment and ensures the ideal moisture environment at the site of an injury. In burns, exudate adsorption should be between 24.05 g/g and 45.65 g/g. This shows that the scaffold is perfectly attached to the injury site and that tissue regeneration is assisted [195]. The architecture of the collagen network allows for the swelling ratio to be adjusted based on the therapeutic needs [196].

### 5.6. Water Vapour Transmission Rate

The WVTR measures the moisture permeability of a scaffold. The WVTR ensures an adequate moist surface surrounds the wound, which is an important component in wound healing. An optimal WVTR range correlates with good exudation drainage at the injury site. The WVTR is distinguished by its collagen derivative. This is because collagen type I derived from fish scale and porcine sources has a WVTR of 952.6 + 55.5 g/m^2^/day and 1090.9 + 77.1 g/m^2^/day, respectively [197]. A low level of the WVTR causes exudate accumulation, whereas a high level of the WVTR causes water to evaporate at the injury site, resulting in dehydration. A WVTR range of 2028.3 ± 237.8 g/m^2^/day is ideal for maintaining a moist environment and enhancing the normal healing phase [118]. The swelling ratio and WVTR depend on the fabrication method used as the design interacts in its own way with the wound environment. For example, gelation polymerisation and lyophilisation can fabricate scaffolds with different porosities, in which hydrogel has less water vapour than sponge as a result of the higher water content in their pores compared to their dry form counterpart. As a result, a wound with high exudation necessitates a higher swelling ratio for the ability of high absorption, which includes foam dressings [198]. In contrast, low exudating wounds are more suitable for the retention ability of a hydrogel dressing [199].

### 5.7. Surface Characteristics

Cell–scaffold surface interaction plays the main role in cell attachment, proliferation, and tissue regeneration [200]. It is an essential factor that governs cell response. Cells tend to attach to hydrophilic surfaces rather than hydrophobic surfaces [201]. The presence of collagen type I provides suitable wettability (hydrophilicity) as the collagen molecule consists of hydrocarbon chains and hydrophilic functional groups [202]. It could be detected via water contact angle measurement. Lowering the contact angle demonstrated a more hydrophilic surface [203]. A contact angle >90° indicates the perfect wettability surface for the collagen type I surface with a good hydrophilicity property.

Generally, the contact angle will be measured at different times to obtain an average value. In this scenario, a high degree of contact angle <90° indicates a low level of the hydrophilic and wettability properties of the scaffold. Meanwhile, the contact angle can be modified using a crosslinking agent such as tannic acid. This is because increasing the concentration of a crosslinking agent will increase the degree of the contact angle by decreasing the hydrophilic property [204]. From here, it can be speculated that the crosslinking agent and porosity greatly affect the degree of the contact angle of collagen type I.

Measurement of the water contact angle (WCA) on acid-soluble collagen (ASC) and pepsin-soluble collagen (PSC) from a collagen type I derivative of red stingray shows significantly different results. This is because the WCA for ASC was 100.3° ± 2.31° with a contact angle of 30.67° ± 1.89°, while for PSC, it was 94.96° ± 0.59°, with a contact angle of 42.00° ± 1.14°. The results indicate that both are hydrophobic (>90°) due to the presence of amino acid and pepsin residues in the collagen [119].

Another feature of the biomaterials’ surface is the roughness, which can influence cellular behaviour and biocompatibility [205]. This is commonly evaluated via AFM to provide the features of biomaterial topography, although energy dispersive X-ray (EDX) can also show peaks and valleys of surface topography and elemental distribution. The adsorption of collagen type I on any surface enhances the surface roughness to allow cells to attach and proliferate. Collagen adsorption has been described to increase the nitrogen and carbon elements on the surface. Yet, nitrogen-associated elements do not influence the hydrophobic property of collagen. It is well known that proteins tend to bind easily to hydrophobic surfaces, and collagen, which binds to the hydrophobic surface, will exhibit reduced resistance to mechanical strength. This is the main reason for the visibility of a patterned structure on the hydrophobic surface [203].

Among the surface characteristics, the evaluation of integrin binding peptide arginine-glycine-aspartic acid (RGD) could be controlled independently to guide cell attachment. Endothelial cell morphology and function are influenced by the physical and chemical properties of the extracellular environment [206]. Several ECM proteins including fibronectin, collagen type I, fibrinogen, laminin, vitronectin, and osteopontin contain the RGD sequence. It is critical to create ECM-like substrates with well-controlled features for studying the effect of RGD on targeted cell behaviour.

In vitro, photocrosslinkable alginate hydrogels containing RGD were able to encapsulate, tether, and retain functionally engineered extracellular vesicles (FEEs) for seven days with maintained structural integrity and osteoinductive functionality. These hydrogels also improved bone regeneration by a factor of four in an in vivo calvaria defect model compared to the controls without the FEEs and by a factor of two compared to the controls lacking the RGD component [207]. This demonstrates that RGD could improve cell attachment.

Silicon surfaces were created to independently control the topographical features and surface densities of RGD. Cell adhesion, spreading, and migration were studied on surfaces with nano- to micro-scaled pyramids and average densities ranging from 6102 to 1011 RGD/mm2. Fewer cells were found to adhere to the rough surfaces compared to the flat surfaces. The optimal average RGD density for cell adhesion on flat surfaces and substrata with nano-scaled roughness was 6105 RGD/mm^2^ [208]. The average RGD density governs the degree of cell spreading and the length of focal adhesion within adherent cells. Although the initial contact of cells with a substrate may be guided by topography, cell surface receptor engagement is primarily controlled by surface chemistry.

### 5.8. Chemical Characteristics of Collagen Type I

The structural and chemical modification of collagen type I to develop a 3D scaffold could change the chemical structure of collagen type I. Thus, its chemical characterization will help to determine the existence of a basic molecule for collagen type I. The common techniques that are used include Fourier transform infrared (FTIR), EDX, and XRD.

#### 5.8.1. X-ray Photoelectron Spectroscopy 

XPS is a common technique used to investigate the surface chemistry of enhanced collagen biomaterials. Scaffolds must have a surface chemistry and microstructure that allow for cell attachment, proliferation, and differentiation. The attachment of cell-binding proteins such as fibronectin is linked to increased hydrophilicity. Surface elemental compositions such as C, N, and O, as well as functional groups such as C-C, C-N, C-O, O=C-N, and O-C=O can be quantified using XPS. Collagen biomaterial surface wettability may become hydrophobic after crosslinking, and a higher content of oxygen-containing functional groups may improve wettability [209]. Increasing oxygen-containing functional groups may be a strategy, called ozone perfusion, to improve surface wettability. The perfusion period is proven to be inversely proportional to the water contact angle of collagen type I. This is because the extension of the perfusion period can alter collagen cleavage. When this occurs, collagen will be split into small peptide fragments. This contributes to excellent cell attachment with a perfect degree of hydrophilicity and wettability without causing any damage to the collagen. XPS will be able to detect successful enhancements in material crosslinking or coating changes. For example, the O=C-N of the pristine scaffold was 118.8%, increased by 13.1% post-crosslinking, and improved by 15.7% after 8 min of treatment with ozone perfusion [209].

XPS makes use of synchrotron radiation, which can detect surface implants and characterise different collagen surfaces [210]. Simultaneously, XPS aids in peak interference by separating overlapping peaks during XPS measurement. According to this statement, an ultra-high vacuum condition is always required during XPS analysis to prevent any means of contamination of the sample. If the sample is not compatible in the vacuum state, it must be cold or at ambient pressure. XPS samples must have a maximum size of 20 cm^2^ and a height of less than 25 mm. XPS analysis of samples reveals a nominal sensitivity of ≈0.1 atomic % with an elemental sensitivity that can vary by up to 100. Meanwhile, a sample size greater than 10 µm will be useful for assessing chemical components [120].

#### 5.8.2. Fourier Transform Infrared

The term Fourier Transform refers to an algorithmic approach for converting the raw data to a spectrum. Infrared (IR) spectroscopy has been used to detect the vibrational characteristics owned by specific functional groups in a material. The functional group will absorb the IR radiation at a specific wavenumber (cm^−1^) range, whereby it represents a chemical bond vibration at a specific frequency [33]. Collagen type I has several functional groups that encompass amides I, II, and III. Furthermore, these amide groups are detected at a range of peak intensities between 1450 cm^−1^ and 1235 cm^−1^, which commonly indicates the helical structure of collagen. These amide groups are not able to differentiate the type of collagen. However, it was reported that the presence of amide A at the higher peak intensity of 3350 cm^−1^ can be attributed to collagen type I [121]. Busra also reported that a peak intensity of 1632 cm^−1^ indicates a higher-order arrangement of the collagen structure referring to the β-sheet and triple helix structure [14]. FTIR is a highly promising technique for obtaining early information on any material, particularly unknown materials.

#### 5.8.3. Energy Dispersive X-ray 

The elemental analysis of materials is important to determine the homogeneity of biomaterial development. EDX is an analytical evaluation used to measure the major element composition of a material. EDX correlates with FTIR, whereby it verifies the major elements that are present in the materials that build a chemical structure to form different functional groups. To date, EDX equipment has a limit on its ability to detect certain elements, especially hydrogen [211]. The major elements in collagen type I are oxygen, nitrogen, and carbon, with oxygen having the highest percentage, followed by nitrogen and carbon [122]. This indicates the purity of the collagen [92], and the elements detected should be similar to those elements contained within the tissue region where this scaffold is going to be implanted. The same ratio of elemental content in collagen to the tissue indicates biocompatibility. This will allow the cells to grow throughout the scaffold [212], and this demonstrates the value of using a collagen type I scaffold in therapeutics.

#### 5.8.4. X-ray Diffraction

The atomic structure of collagen type I can be determined via XRD, which assists in determining whether the materials are in the crystal or amorphous phase [71]. X-ray diffraction results from an electromagnetic wave (the X-ray) impinging on a regular array of scatterers (the repeating arrangement of atoms within the crystal). X-rays are used to produce the diffraction pattern because their wavelength λ is typically the same order of magnitude (1–100 angstroms) as the spacing d between the planes in the crystal form. In principle, any wave occurring on a regular array of scatterers produces diffraction. The interaction of the incident rays with the sample produces constructive interference (and a diffracted ray) when the conditions satisfy Bragg’s law. This law relates the wavelength of electromagnetic radiation to the diffraction angle and the lattice spacing in a crystalline sample. Crystals offer a much stronger signal due to their periodicity compared to the non-crystal sample. A crystalline sample is, by definition, periodic. A crystal is composed of many unit cells repeated indefinitely in three independent directions. In a liquid, powder, or amorphous sample, molecules within that sample are in random orientations. More importantly, the orientational information is lost. Collagen type I demonstrates an amorphous phase instead of crystallinity [71]. Thus, collagen type I has a random arrangement of its atomic structure with inappropriate diffraction and a low captured signal. The XRD of collagen generally consists of two clear peaks where the first peak is sharper than the second peak [71,123,124]. This explains that collagen has ordered structure snippets, and the majority of them are in the amorphous phase [123]. Furthermore, collagen type I from different sources, such as mammalian, avian, marine, fish, and others, via XRD has been proven to be closer to the amorphous phase rather than crystalline.

## 6. Current Insights and Conclusion

### 6.1. Current Insights over the Last Five Years

Over the last five years, the trend of research regarding collagen type I in tissue engineering and regenerative medicine has slightly shifted. The initial stepping stone in building collagen type I as a biomaterial has been laid since this naturally derived polymer has been proven as a safe, biodegradable, and biologically suitable application that has passed a few clinical trials [213]. There are various commercially available three-dimensional collagen-based biomaterials for different applications such as Integra™ (Integra Lifesciences Corporation, New Jersey, USA) for wound healing [214] and Hemocollagene^®^ (Saint-Maur-des-Fossés, France) for dental regeneration [215].

At the beginning, as well as now, many research groups focused on various integrations of natural, as well as synthetic polymers with collagen type I to create a bioscaffold to overcome its original drawbacks of high biodegradability, low mechanical strength, and inductive activity. In addition, there were many ways to manufacture the biomaterial in the form of sponges, films, hydrogels, particles, and others, dependent on the applications. Researchers expanded on new technology to create various forms of scaffolds for different applications such as foam [216] via freeze-drying or organised lattices through electrospinning [217,218]. Some injectable materials, such as hydrogel, are preferred for their applications such as corneal or periodontal injection [219,220]. Scaffolds with collagen type I were also studied for drug delivery mechanisms including nanoparticles [221,222,223]. The modifications of surface wettability, biocompatibility, and antibacterial coatings are important for long-lasting and effective biomaterials.

Such endeavours are still being pursued by scientists today. However, some prefer to integrate collagen with synthetic polymers such as polycaprolactone (PCL), polyglycolic acids (PLGAs) [209], polyvinyl alcohol [224], and glass bead particles [225,226] for various applications. For example, Xu et al. (2022) explored the integration of synthetic polymer polycaprolactone (PCL) with rat tail collagen type I to reduce keratocyte differentiation and prevent fibrosis of the corneal stroma after injury [227]. Peng et al. (2021) used PCL with gelatine and magnesium oxide to create nanocellulose membranes via electrospinning for periodontal tissue regeneration. Nevertheless, it seems that researchers are now moving towards more green technology in tissue engineering by using natural-based sustainable products for their bioscaffolds with collagen including chitosan [228], alginate [229], silk fibroin [230], gelatine [231], starch [232], and a few other, either by themselves or in various combinations. Notably, natural-based materials are not only more sustainable, but they also have higher biocompatibility, improved biodegradability, and in general, little to no toxicity [233]. Furthermore, in the context of the circular economy and sustainability, collagen sources such as bovine hide off-cuts [75], pig skin [82], chicken skin and eggshells [66,68], sheep legs [234], and fish scales [235] can be obtained from food waste. Moving forward, there is a significant need for protocols considering green chemistry such as using and fabricating products that are biodegradable and non-toxic to the environment.

### 6.2. Trends and Future Perspectives

Recently, researchers have been using collagen peptides in their studies. Breaking down the large protein structure into specific collagen-derived peptides seems to target a variety of biological effects on cells. Specific peptides could be used based on the application. For example, collagen-derived hydroxyproline (hyp)-containing peptides are locally generated by the degradation of endogenous collagen in response to injury. Thus, nanostructured collagen–hydroxyapatite composite scaffolds that are doped with magnesium were fabricated to improve biodegradation rates for hard tissue bone regeneration [236]. In addition, dipeptide prolyl-4-hydroxyproline (pro-hyp) exhibits pronounced effects on mouse tendon cells and promotes differentiation or maturation of tendon cells with the modulation of lineage-specific factors and induces significant chemotactic activity in vitro [237]. Peptides are not limited to collagen. For example, recombinant chimeric fibronectin and elastin-like peptides were developed to specifically biofunctionalize titanium surfaces for osseointegration [238]. This concept has been used to bind extracellular vesicles to collagen-derived mimetic peptides for prolonged drug delivery [207], as well as to use antimicrobial peptides as an implant coating to prevent reinfection in patients [239]. The use of collagen-based biomaterials still requires extensive research into cell signalling mechanisms. With more understanding, there is no doubt that research may start to take into account using targeted peptides to improve their application.

Another notable trend in recent years has been the shift away from breaking down collagen or the matrix into functionalised items and toward using the native tissue directly through decellularization. This will theoretically fulfil all of the requirements of a true extracellular matrix by providing topographical and biochemical cues to promote cell attachment and function. Although decellularization is not a new direction in medicine, rapid advancement has been hindered by a multitude of challenges. Transplantation is often the only treatment for organ failure. However, organ scarcity and dependence on immunosuppressive therapy call for acellular scaffolds. These scaffolds are supposed to preserve the architectural and mechanical properties of the original organ, promote cell attachment, growth, and differentiation, as well as pose no threat to the host immune system. Thus far, there has been a significant immune response to allogeneic and xenogeneic transplanted scaffolds [240]. For complex organs, there are limitations on cell differentiation capacity during recellularization including the types of cells used (cell line, primary, stem cells) and the multitude of cell types (vascular, epithelial, interstitial cells, others) required by the recipient. Primary cells have been used successfully on tissue-engineered bladders seeded with autologous primary cells from two different transplantation lineages, with no signs of rejection and full bladder function after a 5-year follow-up [241]. However, some forms of collagen have immunogenic elements on the scaffold. For example, it was suggested that some individuals have antibodies against bovine collagen [242] and that 3% of the population is allergic to bovine collagen [240]. The potential of collagen to induce an immune response is currently being investigated in vitro. However, it was reported that collagen I activates certain types of T-cells and stimulates proinflammatory cytokines. Similar observations were also made with porcine collagen type I [243].

Recent research on decellularization includes simple parts such as small-diameter blood vessel substitutes that are important in vascular diseases. The functional tissue-engineered vascular grafts must be biocompatible, non-immunogenetic, biodegradable for tissue ingrowth, capable of supporting endothelisation, and easily sterilisable with enough mechanical strength to support long-term blood flow. Decellularized matrices and ECM-mimicking bioscaffolds are two methods for achieving this [244]. Decellularized extracellular matrix hydrogels, which may or may not contain cells, can be used as printing inks for three-dimensional bioprinting [245,246,247]. Other decellularization processes include decellularizing cornea to fabricate improved constructs [248], decellularizing human-umbilical-vein-endothelial-cell (HUVEC)-derived extracellular matrix to be integrated into a manufactured scaffold with collagen type I to improve endothelial cells with progenitor cell crosstalk due to their intact angiocrine biomolecules for osseointegration [249], and decellularizing rat livers to form acellular 3D bioscaffolds suitable for seeding with induced pluripotent stem cells (iPSCs) [250]. The focus of research may vary depending on the technology and application, but the fundamental use of collagen type I is undeniable.

### 6.3. Conclusions

The sources of collagen I, various extraction methods, molecular structure and biosynthesis processes, and methods for physicochemical characterisation of collagen type I were all covered in this review. Understanding biosynthesis and molecular structure provides a solid foundation for justifying the use of various extraction methods. The extraction procedure ultimately influences the yield and integrity of the resulting collagen polypeptide chains, as well as the functional properties such as viscosity, water retention, solubility, and emulsification. These could all differ depending on the processing parameters such as time, temperature, enzyme and pH levels, pre-treatment method, storage process, and raw material properties. Consistent production of high-quality collagen is critical for reaping its benefits in future therapeutic and industrial applications, particularly in mass production. The distinct properties of collagen make it suitable for modification and incorporation in a variety of fields, particularly therapeutic applications. Although collagen from different sources has different physicochemical properties, they are all generally triple helical polypeptides that can be manipulated, and the raw materials can be obtained from by-products, making it more sustainable. In this context, having a general understanding of the synthesis, structure, source, extraction process, and validation procedures used to characterise collagen properties is of great interest and adds value to standardising the use of collagen type I in various fields.

## Figures and Tables

**Table 1:** Some extraction procedures used for collagen type I from different sources.

Animal Class	Source and Tissue Type	Pre-Treatment Method	Extraction and Purification Method	Scaffold Comparisons	Reference
Mammalian	Human-placenta	Alkaline; 0.1 M NaOH in 1:10 (*w*/*v*) ratio; 6 h stir	Acid; 0.06 M acetic acid 1:25 (*w*/*v*) overnight; NaCl 3 M pH 7 to collect supernatant; dialysis against distilled water 5 days	-Cost effective (human waste) -Safe and fast extraction method	Karami et al., 2019 [91]
	Bovine skin, bone, pericardium	Alkaline; 300 mL of 0.1 M NaOH at 4 °C 48 h; filtered 3X	Acid–enzyme; 300 mL 10 mM HCl with pepsin to solution ratio of 1:20; pH 2.0 4 °C 12 h; urea and NaCl to collect supernatant; 1 M Tris for pepsin deactivation; dialysis against 1 M phosphate buffer 4 °C 16 h	-Collagen in the purified form -Can be a substitute/replaced from the commercial collagen -Simple extraction method	Santos et al., 2013 [92]
	Rat tail	Salt; 10% NaCl 4 °C 24 h	Acid–enzyme; 0.5 M HCl and pepsin 1:50 4 °C 24 h; NaCl to collect supernatant and washed with phosphate buffer pH 7.4, 0.02 M	-Biocompatible -Induce contraction effect in in vitro model -Excellent for dental application	Techatanawat et al., 2011 [93]
	Goat tendon	Acid; 1% (*v*/*v*) acetic acid 4 °C 72 h	Acid; 1% *v*/*v* after cotton mesh filtration and dialysis against 10 mM PBS 48 h, then further dialysed against 0.05 M Na_2_ HPO_4_	-In vitro and in vivo study towards HUVEC cells showed prominent result -Good for future angiogenesis study	Banerjee et al., 2012 [94]
	Sheep tendon	Acid; 0.35 M acetic acid 4 °C 24–48 h	Acid; 0.35 M acetic acid; NaCl salting-out 4 °C 24–48 h; supernatant undergoes dialysis against Na_2_HPO_4_ and PBS 14 kDa 4 °C 72 h	-Biocompatible toward human dermal fibroblasts (HDFs) -Greater availability and wider acceptability (religious views) -Can be fabricated into porous scaffolds	Fauzi et al., 2016 [71]
Fish	Freshwater fish scales	Salt; 1.0 M NaCl, 0.05 M Tris HCl, 20.0 mM EDTA 48 h (pH7.5); demineralisation 0.5 M EDTA 48 h (pH 7.4)	Acid; 0.5 M acetic acid (pH 2.5) 48 h; NaCl (0.9 M) salting-out 24 h; resolubilised supernatant undergoes dialysis against 0.1 M acetic acid and deionised water 24 h	-Cost effective -Alternative to other collagen sources -Highly biocompatible	Pati et al., 2012 [95]
	Bighead carp fins, scales, skin, bones, and swim bladders	Alkaline; 0.1 M NaOH 1:10 (*w*/*v*) ratio; 4 °C 36 h	Acid–enzyme; 0.5 M acetic acid 0.1% (*w*/*v*) pepsin 1:10 (*w*/*v* days; NaCl to collect supernatant 2 M; dialysis against distilled water 7 kda	-Alternative to other sources of collagen -Pepsin-solubilized collagen (PSC) can be extracted	Liu et al., 2012 [96]
	Loach skin	Alkaline–salt; 10% NaCl 1:5 (*w*/*v*) 4 °C 1 h. 0.1 mol/L NaOH 1:10(*w*/*v*) 4 °C 24 h	Acid–enzyme; 0.5 mol/L acetic acid 1:30(*w*/*v*) 4 °C 24 h 5% (*w*/*v*) pepsin 2X; NaCl 0.9 mol/L to collect supernatant; dialysis against distilled water 8 kDa	-Alternative to other sources of collagen -Acid-soluble collagen (ASC) and pepsin-soluble collagen (PSC) can be extracted	Wang et al., 2018 [97]
	Clown featherback skin	Alkaline; 0.1 M NaOH 1:15 (*w*/*v*) 4 °C 8 h	Acid–ultrasound; 0.5 M acetic acid 4 °C 48 h; in 30 min ultrasonication (20–80% between 10–30 min); NaCl to collect supernatant to 2.6 M; dialysis against distilled water for 2 days	-Ultrasonic application improves the extraction efficiency -Higher yield production, alternative to other collagen sources	Petcharat et al., 2020 [89]
Marine	Jumbo squid mantles	Acid; 6 M urea in sodium acetate (pH 6.8) and neutral buffer 24 h	Acid; 0.5 M acetic acid 24 h; supernatant collected for acid soluble collagen	-Acid-soluble collagen (ASC) can be extracted -Have some similar characteristics to bovine collagen	Uriarte-Montoya et al., 2010 [98]
	Jellyfish tissues	Acid; 0.5 M acetic acid	Acid–enzyme; 0.5 M acetic acid centrifuged and added pepsin (1–15 mg/mg tissue) overnight; NaCl (0.9 M) used to collect supernatant and dialysed against 0.1 M acetic acid	-Alternative to other sources of collagen -Biocompatible in in vitro testing	Addad et al., 2011 [99]
	Jellyfish and squid	Acid; 0.5 M acetic acid 1:15 (*w*/*v*) 4 °C 3 days	Acid; 0.5 M acetic acid 1:15 (*w*/*v*) 4 °C 3 days after filtration through cheesecloth; NaCl (0.9 M) and 0.05 M Tris and used to collect supernatant and dialysed against 0.1 M acetic acid 3 days and distilled water another 3 days	-Alternative to other sources of collagen -Pepsin-soluble collagen (PSC) can be extracted	Jankangram et al., 2016 [100]
Amphibian	Bullfrog fallopian tubes	Alkali; 0.1 M NaOH 4 °C 1 day	Acid–enzyme; 0.5 M acetic acid with 10% pepsin 4 °C 2 days; NaCl (0.7 M) to collect supernatant; resolubilised acid solution dialysed against 0.1 M acetic acid 1 day; distilled water 2 days	-Pepsin-soluble collagen (PSC) can be extracted -Potential alternative and supplements from other sources of collagen	Wang et al., 2011 [101]
Avian	Skin, skin dermis	Acid (acetic acid)	Acid-based extraction (acetic acid); enzymatic method (pepsin)	-Non-immunogenic and non-allergenic -Biocompatible -Can be fabricated into porous scaffolds	Peng et al., 2010, Parenteau-Bareil et al. 2011 [102,103]
	Chicken cartilage	Acid (EDTA)	Salting-out (NaCl) + ultrasound	-Increased yield production through ultrasound -Pepsin-soluble collagen (PSC) can be extracted	Akram and Zhang, 2020 [88]
	Chicken lungs	Acid salt (sodium carbonate)	Acid extraction (acetic acid) + salting-out (NaCl) + enzymatic method (pepsin) + ultrasound	-Increased yield production through ultrasound -Alternative source to mammalian collagen -Higher thermal stability	Zou et al., 2020 [87]

**Table 2:** Collagen type I physicochemical characterisation methods and expected results.

Characterisation	Method	Expectation	Reference
**Biocompatibility**			
Biocompatibility and immunogenicity	MTT assay Live/dead assay Cell attachment Immunocytochemistry (ICC)	Cell proliferation and growth; SEM; more than 80% cell adhesion after 24 h; integrin-related protein expression and cell attachment	Addad et al., 2011 [99], Fauzi et al., 2016 [71], Fauzi et al., 2017 [104], Thievessen et al., 2015 [105]
**Physical, Morphological, and Topographical (PMT) Characterisation for Three-dimensional stability**			
Weight	SDS-PAGE	Type I collagen is composed of β (250 kDa), α_1_ (130 kDa), and α_2_ (115 kDa)	Peng et al., 2010 [102], Parenteau-Bareil et al., 2010 [106], Fauzi et al., 2016 [71] Inanc et al., 2017 [107]
Native conformation	UV-circular dichroism (CD) spectroscopy	CD with a positive maximum absorption band at around 222 nm	Carvalho et al., 2018 [108]
Mechanical strength	Tensile strength and Young’s modulus	No gold standard, but fish and reptile should be more fragile than mammalian material	Amri et al. 2014 [109], Teramoto et al. 2012 [110]
Thermal stability	Thermogravimetry analysis (TGA) Differential scanning calorimetry (DSC)	No gold standard for what T_d_ of biomaterial should be, but it must be stable to use normal temperatures or higher; in general, comparable or higher than native rat tail tendon collagen fibre T_d_ of 65 °C; T_d_ of extracted collagen in solution are 37–40 °C, 26- °C, and 6–20 °C, for mammalians, fish, and deep-sea animals, respectively	Miles and Bailey 1999 [111], Zhang et al., 2020 [112], Subhan et al., 2015 [113], Bozec and Odlyha, 2011 [114]
Porosity and pore size	SEM	Pore size > 80 µm for fibrogenic and <20 µm for chondrogenic growth; the average mean size for ovine-, bovine-, and porcine-derived scaffolds are 73.05 ± 10.79 µm, 85.84 ± 9.51 µm, and 87.32 ± 10.69 µm, respectively	Ghodbane and Dunn 2016 [115]
Biodegradation	Enzymatic biodegradation method	Depends on application example within 14 days for cutaneous wound healing	Mh Busra et al., 2019 [116] Salleh et al., 2022 [117]
Swelling ratio	Swelling ratio protocol	1000–2700% for biomaterials	Ghodbane and Dunn 2016 [115]
Water vapor transmission rate (WVTR)	WVTR method	Within range 2028.3 ± 237.8 g/m^2^/day to maintain a moist environment and enhance the normal healing phase	Xu et al., 2016 [118]
Surface and particle physicality	Contact angle zeta potential	Angle < 90° hydrophilic; isoelectric points (pIs) close to 6 at zero zeta potential	Chen et al., 2019 [119]
**Chemical Characterisation**			
X-ray photoelectron spectroscopy (XPS)	XPS	Samples should show ≈0.1 atomic % as nominal sensitivity with an elemental sensitivity that may differ as much as ≈100; for the assessment of chemical components, a sample size larger than ≈10 μm will be convenient	Baer et al., 2019 [120]
Fourier transform infrared (FTIR)	FTIR	Collagen type I functional groups include amides I, II, and III; range of peak intensity between 1450 cm^−1^ and 1235 cm^−1^ commonly indicates the helical structure of collagen; amide A at the higher peak intensity of 3350 cm^−1^ can be attributed to collagen type I; At peak intensity of 1632 cm^−1^, this indicates the higher-order arrangement of the collagen structure, which refers to β-sheet and triple helix structure	Sasmal and Begam 2014 [121] Fauzi et al., 2016 [71]
Energy dispersive X-ray (EDX)	EDX	Major elements in collagen type 1 are oxygen, nitrogen, and carbon with a higher percentage of oxygen, followed by nitrogen and carbon	Fauzi et al., 2014 [122]
X-ray diffraction (XRD).	XRD	Collagen XRD generally consists of 2 clear peaks, where the first peak is sharper than the second peak; collagen type I from different sources of mammalian, avian, marine, fish, etc., via XRD has been proven closer to the amorphous phase rather than crystallinity	Zhang et al., 2011 [123], Fauzi et al., 2016 [71], León-Mancilla et al., 2016 [124]

**Table 3:** Main methods and classification of crosslinkers according to [19,112,142].

Table		Mechanism	Main Characteristics	
			Pros	Cons
Physical	Dehydrothermal treatment (DHT)	Water is removed as amide bonds are formed between amine and carboxyl groups of collagens (inter-molecular). Lysino-alanine bonds are also formed between lysine residues and dehydro-alanine in collagen chains (intra-molecular) [112,144].	Safe. Non-toxic to cells. Thermally stable. Sterilization.	May cause denaturation. Low collagenase resistance [153].
	Ultraviolet irradiation (UV)	Non-ionizing irradiation to induce collagen amino acids residues—Trp, Phe, and Tyr—to generate free radicals to cause inter-molecular crosslinks [146].	Rapid process. Non-toxic to cells. Sterilization.	May cause denaturation. Poor crosslinking strength.
	Gamma ray/electron beams	Free radical mediation—hydroxyl radicals from amino acid residues—Phe, His, and Met—form intermolecular crosslinks [164]. Furthermore, two tyrosyl radicals can form dityrosine bonds and crosslink [165].		
Chemical	Glutaraldehyde (GA)	Aldehydes crosslink collagen by forming Schiff bases between the ε-amino groups from lysine or hydroxylysine residues and the aldehyde groups of aldehyde [112].	Low cost. High reactivity. High water solubility.	Potential cytotoxicity to cells.
	Dialdehyde starch		Low cytotoxicity. Biodegradable. Resistant to collagenase.	Costly. Sensitive to oxidation [166].
	Epoxy compounds	Epoxy groups form multipoint crosslinking with ε-amino groups of lysyl residues at alkali condition [167].	Makes tissues more pliable [168]. Biocompatible with corneal cells [152].	Affected by pH [167].
	N-hydroxysuccinimide (NHS)	Carboxyl groups from polycarboxylic acids are turned into active ester groups, which then crosslink with free amino groups within different collagen chains [147].	Highly resistant to collagenase [142].	Requires thorough washing.
	1-ethyl-3(3-dimethylaminopropyl)-carbodiimide (EDC)	Crosslinking via amido bond between carboxyl groups and amino groups in adjacent collagen chains. EDC activates carboxyl groups (Glu and Asp) into active O-acylisourea groups, which undergo nucleophilic attack by free amine groups (lys or hyl), forming amido bonds. Usually, NHS is added to catalyse the reaction (EDC/NHS crosslinking) [169,170].	Stronger crosslinking effect. Zero-length crosslinker and intra-fibrillar. Low cytotoxicity.	Can cause excessive crosslinking and stiffness.
	Diphenylphosphorylazide (DPPA)	Converts carboxyl groups into acylazide groups, which react with the free amino groups of adjacent collagen chains (amido bonds) [148].	Fast. Non-toxic. Resistant to collagenase.	Difficult to remove solvent.
Biological	Genipin	Crosslinking by initiating a nucleophilic attack on amine with two amino groups. The genipin C3-atom undergoes nucleophilic attack from primary amine groups (lys, hyl, arg), which then reacts with another collagen amino group by replacing an oxygen on the genipin ring, forming a nitrogen–iridoid (heterocyclic compound). Two iridoids are linked through a radical reaction to form intramolecular and intermolecular crosslinks [155,156]	Natural resources, biocompatible with cells. Resistant to collagenase.	Excessive concentration will reduce scaffold mechanical strength. Blue reaction after crosslinking. Sensitive to light and oxidation.
	Transglutaminase (TG)	Catalysed γ-carboxamide groups of gln residues become the acyl donor for acyl receptors, which are the ε-amino groups of lys residues or primary amino groups. Then, ε-(γ-glutamyl) lysine bonds form intra- and intermolecular bonds [158].	Non-toxic, good biocompatibility. Similar to natural crosslinking.	Enzyme deregulation may cause harmful diseases.
	Plant polyphenols/procyanidin/proanthocyanidins (PA)	Multipoint hydrogen bonds formed between phenolic hydroxyl groups of polyphenols and hydroxyl, carboxyl, amino, or amide groups of collagen chains [162]	Very low cytotoxicity. Antioxidants. Anticancer, antimicrobial, antiangiogenic, and anti-inflammatory [163].	Sensitivity to thermal treatment, light, and oxidation.

## Data Availability

Not applicable.
